# In Silico Investigations of Multi-Drug Adaptive Therapy Protocols

**DOI:** 10.3390/cancers14112699

**Published:** 2022-05-30

**Authors:** Daniel S. Thomas, Luis H. Cisneros, Alexander R. A. Anderson, Carlo C. Maley

**Affiliations:** 1 Arizona Cancer Evolution Center, Arizona State University, Tempe, AZ 85287, USA; daniel.saha@asu.edu (D.S.T.); lhcisner@asu.edu (L.H.C.); 2 School of Life Sciences, Arizona State University, Tempe, AZ 85287, USA; 3 Biodesign Center for Biocomputing, Security and Society, Arizona State University, Tempe, AZ 85287, USA; 4 Integrated Mathematical Oncology Department, Moffitt Cancer Center, Tampa, FL 33647, USA; alexander.anderson@moffitt.org; 5 Biodesign Center for Mechanisms of Evolution, Arizona State University, Tempe, AZ 85287, USA; 6 Center for Evolution and Medicine, Arizona State University, Tempe, AZ 85287, USA

**Keywords:** adaptive therapy, cancer, drug resistance, dose modulation, evolution, agent-based model

## Abstract

**Simple Summary:**

Modern “adaptive therapy” approaches to cancer therapy rely on adjusting the dose of drugs as the size of the tumor changes. They hold the promise of transforming cancer from an acute lethal disease to a chronic disease we could live with, but not die from. Previous adaptive therapy experiments have used a single drug. We set out to explore how to best combine multiple drugs in these strategies. Unfortunately, there are far too many possible ways we might combine drugs in adaptive therapies to be evaluated with clinical trials. Instead, we used computer simulations of how cancers evolve in response to therapies to identify the most promising strategies that should be tested in mouse experiments and in clinical trials in the future. These promising strategies were not specific to any particular drug or particular type of cancer, and so may have general applicability for virtually all cancers.

**Abstract:**

The standard of care for cancer patients aims to eradicate the tumor by killing the maximum number of cancer cells using the maximum tolerated dose (MTD) of a drug. MTD causes significant toxicity and selects for resistant cells, eventually making the tumor refractory to treatment. Adaptive therapy aims to maximize time to progression (TTP), by maintaining sensitive cells to compete with resistant cells. We explored both dose modulation (DM) protocols and fixed dose (FD) interspersed with drug holiday protocols. In contrast to previous single drug protocols, we explored the determinants of success of two-drug adaptive therapy protocols, using an agent-based model. In almost all cases, DM protocols (but not FD protocols) increased TTP relative to MTD. DM protocols worked well when there was more competition, with a higher cost of resistance, greater cell turnover, and when crowded proliferating cells could replace their neighbors. The amount that the drug dose was changed, mattered less. The more sensitive the protocol was to tumor burden changes, the better. In general, protocols that used as little drug as possible, worked best. Preclinical experiments should test these predictions, especially dose modulation protocols, with the goal of generating successful clinical trials for greater cancer control.

## 1. Introduction

Historically, the standard treatment (ST) for most solid tumors has been the maximum tolerated dose (MTD) of a cancer drug [1,2], with the ultimate goal of eradicating the tumor. However, advanced cancers often quickly evolve drug resistance under ST protocols. Cells in a cancer are heterogeneous [3,4,5,6,7,8,9,10] and constant application of high doses of drugs eliminates drug sensitive cells while drug resistant cells survive, divide, and multiply, taking over the tumor [11,12,13]. In ecology, this is called competitive release [14]. The same phenomenon occurs in the evolution of pesticide-resistant pests when treated with high doses of pesticides [15,16]. However, resistance to treatment typically comes at a fitness cost [17]. That is, resistant cells face a penalty, which can be measured in increased doubling times relative to the sensitive cells [17]. In vitro competition experiments using a co-culture of drug sensitive MCF7 and drug-resistant MCF7Dox cell line have demonstrated that, in the absence of the drug, MCF7 cells can outcompete MCF7Dox cells within a few generations [17]. Inspired by pest management, Gatenby and colleagues have shown that robust cancer control is possible if there is a substantial fitness cost to resistance [18]. Adaptive therapy leverages the fitness cost of resistance, using competition with sensitive cells to keep resistant cells under control [1,14,17,18,19,20,21,22,23,24,25,26,27,28,29,30]. This can result in long-term containment of the tumor, especially suitable for cases where a straightforward cure is not attainable due to the presence of drug-resistant cells at diagnosis.

Recent preclinical experimental studies in mice with ovarian and breast cancer cell lines have demonstrated the superiority of dose modulation adaptive therapy over MTD treatment in maintaining a stable tumor burden and increasing time to progression [19,20]. A clinical trial of adaptive therapy for metastatic castrate-resistant prostate cancer patients has extended the median time to progression to at least 27 months compared with a contemporaneous study of prostate cancer patients having a median time to progression of about 16 months with standard of care treatment [21]. Theoretical models have been useful for exploring the dynamics of cancer and novel approaches to therapy [31,32,33,34,35,36,37,38,39,40]. Computational simulations using an agent-based model provide evidence that dose modulation adaptive strategies are superior in controlling cancer compared with an MTD approach, especially when the tumor is heterogenous [17]. Optimal control theory has also been used to develop adaptive therapy protocols [41]. Mathematical models have shown that adaptive therapy can work even when there is no fitness cost of resistance under some conditions (e.g., high turnover) [42,43].

Most of the empirical and theoretical studies on adaptive therapy have focused on single drugs [17,19,20,21,25], whereas most chemotherapy protocols for cancer involve multiple drugs [44,45,46,47]. Can we improve on adaptive therapy protocols by using multiple drugs? Adaptive therapy protocols already involve more variables than standard, fixed dose protocols, and the addition of multiple drugs leads to a combinatorial explosion in the number of possible adaptive therapy protocols. It is, therefore, impractical to test many different protocols in mice. Mathematical and game theory models for multi-drug adaptive therapy have previously been developed to find the best way to combine two drugs to treat metastatic castrate-resistant prostate cancer [22,23,29]. Mathematical control theory has also been used to formulate a multidrug regimen for leukemia [48]. However, these previous modeling efforts either do not include spatial structure [22,23], which can have dramatic effects on the clonal competition in cancers [25], or their results depend, in part, on the cost of resistant cells allowing sensitive cells to encapsulate them and thereby prevent resistant cells from proliferating [17,25]. More recent work from Strobl and colleagues [25,49,50] explicitly quantifies this spatial competition between sensitive and resistance cells.

Our main goal here was to explore how we might best combine drugs in adaptive protocols to prevent therapeutic resistance from spreading in a tumor. In addition, we wished to address some caveats of previous models and test how different types of spatial constraints affect adaptive therapy protocols. Moreover, traditional differential equation models of multi-drug adaptive therapy do not capture these spatial interactions which can, nevertheless, play an important role and affect treatment outcome in important ways. In this study, we modelled multi-drug adaptive therapy with two drugs, exploring five different adaptive therapy protocols, and the standard treatment (ST), at maximum tolerated dose until progression, using a hybrid agent-based model [51], under different assumptions of spatial constraints on clonal competition. We investigated protocols that either applied both drugs at the same time, or alternated them in some fashion, by either adjusting the dose or using a fixed dose with some form of drug holiday. Our primary outcome was time to progression, which was defined by the average tumor burden exceeding a threshold set by the control condition of no treatment, or the proportion of cells that were resistant to both drugs expanding to such a significant size (20% of the simulated space) that the protocol was doomed to failure. In this way, we sought to identify multi-drug adaptive protocols that could achieve long-term control of therapeutic resistance.

## 2. Materials and Methods

We described the details of our agent-based model using the standard overview design details (ODD) format from Grimm et al. [52]. We implemented our model by utilizing the Hybrid Automata Library (HAL), which is a hybrid agent-based modeling framework designed to model discrete cell agents interacting with continuous chemical dynamics [51].

### 2.1. Purpose

Our goal was to determine how to combine drugs in an adaptive therapy protocol in order to increase time to progression (TTP), and hopefully prevent progression altogether.

### 2.2. Entities, State Variables, and Scales

We modelled a tumor as a collection of interacting cells located on a 100 by 100, 2-dimensional square lattice. Only one cell could occupy a lattice location at a time, and cells were restricted to being only on the lattice. We used this relatively small cell population size due to computational constraints. While this was smaller than is realistic for even a small tumor [53], we compensated by using a high mutation rate, making the tumor far more difficult to cure with chemotherapy. For the 2-drug regimen, there were four cell types: doubly sensitive cells (sensitive to both drugs), cells resistant to drug 1, cells resistant to drug 2, or doubly resistant cells (resistant to both drugs).

### 2.3. Process Overview and Scheduling

**Figure 1:**
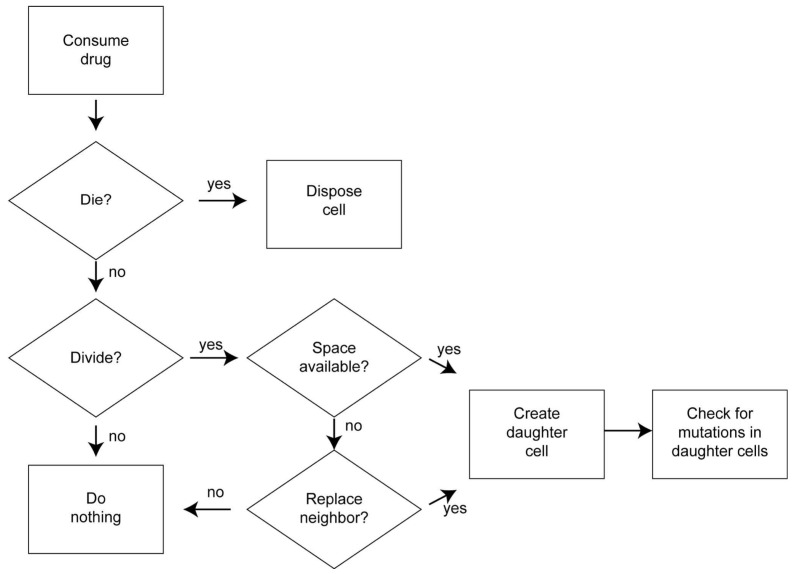
Process overview and scheduling. Cells die as a function of their sensitivity to the drugs, the available drug concentrations and the background death function. A cell divides as a function of its doubling time (resistant cells have slower doubling times). The effects of cell crowding, cell cannibalism and contact inhibition are represented by a probability of replacing a neighbor if there is no open space.

**Figure 2:**
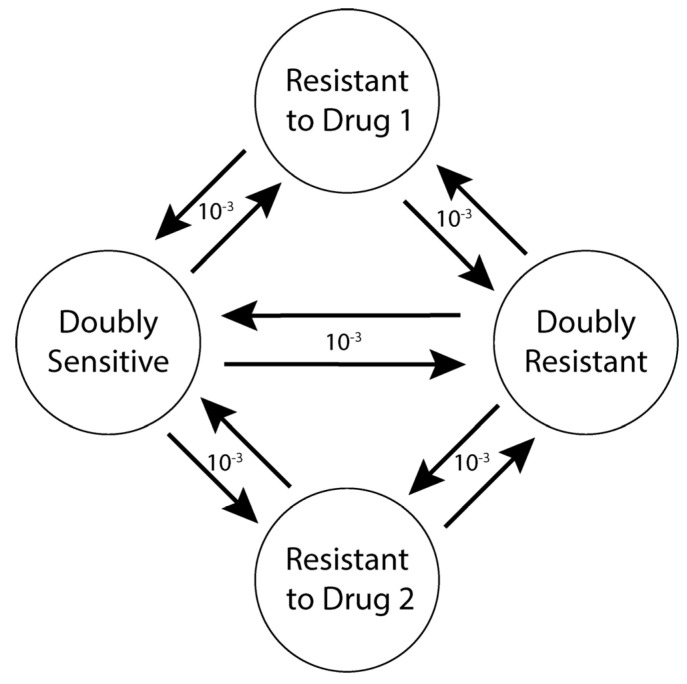
Mutation Schematic. A given cell type can mutate to any other cell type but itself with an equal probability of 10^−3^ per cell division. Doubly sensitive cells can mutate to become doubly resistant cells in one step (e.g., due to multiple drug resistance mechanisms [38]) or via an intermediate step of singly resistant cells. Resistant cells can also mutate to become sensitive again. This may represent epigenetic forms of resistance that are easily reversible.

At every time step, the scheduler (Figure 1) updated the local drug concentration in each lattice site, iterating over each cell in a random order. Whether the cell survived or died depended on cell death probability (Section 2.7.1: Cell Death), which depended on both the background death probability and the probability of death due to drug treatment. If the cell survived, it might divide, subject to the division probability of the particular cell type (Section 2.7.2: Cell Division) and whether or not space was available in the adjacent Moore neighborhood. If space was available, the cell divided, creating a daughter cell, randomly placing it in one of the available spaces in its Moore neighborhood. However, if no space was available, the dividing cell might be able to replace a neighboring cell depending on the replacement probability (Section 2.7.3: Competition for Space). Daughter cells could mutate every time a cell underwent division (Section 2.7.4: Mutation). Doubly sensitive cells could mutate to become doubly resistant cells in one step or via an intermediate step by becoming a singly resistant cell. Cells could mutate in both forward and reverse directions (Figure 2).

### 2.4. Design Concepts

#### 2.4.1. Basic Principles

The basic principles are: (1) tumors are heterogeneous with respect to sensitivity and resistance to drugs; (2) competition between different cell types in tumors is local, between neighboring cells; and (3) drug dynamics occur over continuous space and at a faster time scale than cell division. These features of cancer therapy make a hybrid agent-based model ideal for capturing the dynamics of cancer evolution in response to therapy.

#### 2.4.2. Emergence

We observed clonal dynamics and in particular, the evolution of therapeutic resistance. We routinely observed competitive release in addition to selection for different cell types.

#### 2.4.3. Adaptation

Cells in the model evolved a resistance adaptation to the drugs, encoded in the four possible cell types.

#### 2.4.4. Objectives

The fitness function for the agents was implicit in the model. Different cells had different division and death rates (probabilities per unit time). Natural selection, and selection due to the cancer drugs, acted upon those phenotypes.

#### 2.4.5. Learning

Not applicable.

#### 2.4.6. Prediction

Not applicable.

#### 2.4.7. Sensing

Cells could be killed by the amount of drug in their local environment, but they did not make any decisions based on sensing that concentration.

#### 2.4.8. Interaction

Cells interacted with one another directly when they divided and replaced a neighboring cell (see Section 2.7.3: Competition for Space).

#### 2.4.9. Stochasticity

Stochasticity was an important feature of our model as cell death, division, mutation and replacement are stochastic processes. For each protocol with a given set of parameter values, we ran the model at least 50 times to account for this stochasticity.

#### 2.4.10. Collectives

There were no collectives in the model.

#### 2.4.11. Observation

We ran the model for 5000 time steps (approximately 208 days), with each time step representing 1 h. We determined whether the tumor had “Progressed” or was “Controlled”, and we also recorded the time at which the tumor “Progressed”. Progression was defined by the following survival criteria: if the rolling average of tumor burden over a period of 500 time steps equaled or exceeded 98% of the carrying capacity, or if the rolling average of the total number of doubly resistant cells over a period of 500 time steps equaled or exceeded 20% of the carrying capacity, then the particular run was scored as “Progressed” and the time at which the progression took place was noted. We included the percent of resistant cells in our progression criteria because they were the clinically important population that could not be controlled medically and if they became common would eventually lead to an uncontrollable tumor. The 20% threshold for the doubly resistant cells was a somewhat arbitrary choice to identify cases where the doubly resistant population had started to grow out of control. However, we never observed cases where that doubly resistant population could be controlled above that threshold. Every run was replicated 50 times with a seed that was based on the clock, and we generated a summary of those runs with Kaplan–Meier curves, and analyzed the results with Cox proportional hazard regressions, which calculated the hazard ratio (HR). The HR was the chance that the tumor would meet the progression criteria in the next time interval in the experimental group (e.g., an adaptive therapy protocol) compared with some control group (usually the standard treatment protocol).

### 2.5. Initialization

Parameters were set at the start of a model run depending on user input or an input file (default values are shown in Table 1). Tumor cells were seeded, approximately at the center of the grid, in the form of a disc of radius 10 units, containing about 400 cells, such that each cell had an equal probability of being assigned to be one of the four cell types (Section 2.2). We seeded the random number generator from the clock for every run of the model.

### 2.6. Input Data

Not applicable.

### 2.7. Submodels

#### 2.7.1. Cell Death

Probability of cell death in a time step (1 h) was the sum of the background death probability and probability of death due to individual drugs. Every cell type had the same intrinsic background death rate set at 0.01 per hour, for the default parameters. The equation of probability of cell death for a 2-drug regimen was as follows: probability of cell death per hour = background death probability per hour + S1*[Drug1]*Ψ1 + S2*[Drug2]*Ψ2, where S1 and S2 are binary indicator variables for the cell’s sensitivity to drugs 1 and 2, respectively, such that a value of 1 indicates sensitivity, and a value of 0 indicates resistance to the particular drug; [Drug1] and [Drug2] are the concentration of those drugs (non-negative real values); and Ψ1 and Ψ2 are the drug potency (non-negative real values) of the corresponding drug, quantified as the probability of cell death per unit drug concentration per hour. The probability of cell death never exceeded 1 in any of our parameter settings.

#### 2.7.2. Cell Division

As long as a cell did not just die, it had a chance to divide each time step, determined by the cell division rate of the particular cell type (Table 1). To incorporate the fitness cost of resistance in our model, division probabilities for the cell types were arranged in decreasing order, as follows: doubly sensitive cells > resistant to drug 1 = resistant to drug 2 > doubly resistant cells. Thus, the doubly sensitive cells had the highest, singly resistant cells to either drugs had intermediate, and doubly resistant cells had the lowest cell division rates.

#### 2.7.3. Competition for Space

If there were no empty spaces adjacent to a dividing cell, it might replace one of its neighbors, with a probability set by the replacement probability parameter. This was a computationally efficient abstraction to deal with a gap in the scientific literature. We do not know the relationship between cell crowding and cell death. Some level of cell crowding must kill cells. There is evidence that crowding can collapse local capillaries [54,55]. Modeling capillary dynamics or elastic tissues, where cells could push aside neighbors, is computationally expensive. We do know it is common that cancer cells consume their neighbors [56,57]. Contact inhibition is also a common phenomenon where the presence of neighbors inhibits cell division [58,59]. Setting different values of the probability (*r*) for replacing a neighbor can represent a spectrum of behavior, from contact inhibition (*r* = 0) to neighbor killing (*r* = 1), with neighbor death due to crowding in the middle.

#### 2.7.4. Mutation

The possible transitions between cell types are shown in Figure 2. These transitions represent mutations that occur at a constant rate determined by the mutation rate parameter (the default is 10^−3^ per cell division, Table 1). We chose this particular value so as to make the effective mutation rate comparable to real tumors [53], where tumor burden commonly approaches a billion or more cells. Moreover, a high mutation rate ensured that doubly resistant cells were constantly being generated during our simulations, thus limiting treatment protocols that worked just because the doubly resistant cell population was small. The high mutation rate and the ability to interconvert between the cell types may also represent epigenetic mechanisms of therapeutic resistance [60,61].

#### 2.7.5. Drug Dynamics (Diffusion and Metabolism)

Drugs were applied once a day (the frequency of drug application parameter) for one hour (drug on time parameter; Table 1). We made drug delivery uniform throughout the lattice such that each lattice site received the same amount of drug, representing a well perfused tumor. A fraction (10%, Table 1) of each drug decayed at every time-step. Cells were exposed to the remaining drug which could also freely diffuse into neighboring lattice sites. Drug diffusion was modeled using the alternating direction implicit (ADI) method [51].

#### 2.7.6. Drug Protocols

For all adaptive therapy protocols explored here, dose modulation (DM) and fixed dose (FD), tumor burden was monitored every 3 days. We modeled error in the tumor burden measurement by adding noise (drawn from a Gaussian distribution with a mean of the current tumor burden and a standard deviation of 5 cells). We initiated treatment at MTD as soon as the tumor burden equaled or exceeded 50% of carrying capacity (5000 cells).

For the DM protocols, starting with MTD, dosage of the drugs was increased by the Delta Dose parameter if the tumor grew above the Delta Tumor threshold, and the dosages were decreased by the Delta Dose parameter if the tumor shrank by at least the Delta Tumor threshold. If the tumor grew by no more than the Delta Tumor threshold or shrank less than the Delta Tumor threshold, then the same drug dosage was administered in the next cycle. We also took the absolute tumor burden into consideration (called maximum tolerable tumor burden). If the current tumor burden exceeded the maximum that had been recorded so far, then the dosage was increased by Delta Dose. This occurred so as to prevent the tumor from growing progressively below our ‘Delta Tumor’ threshold for consecutive treatment cycles. In addition, if the tumor burden ever fell to, or below, the “stop dosing” threshold (which defaulted to 2500 cells), a treatment vacation was triggered, during which no drug was administered for the treatment cycle. This modelled the common clinical practice of stopping treatment if the tumor burden falls below detectable levels. Finally, we assumed that it may be difficult to formulate a cancer drug at very low dosages, so we set a minimum drug dose. If a DM protocol would cause the dose to fall below that level, we kept the dose at the minimum dose.

For the FD protocols, drug dosage was set at 75% of the MTD with a cocktail formulation, to match the experiments in [20]. FD Intermittent relied solely on the value of the absolute tumor burden.

**Figure 3:**
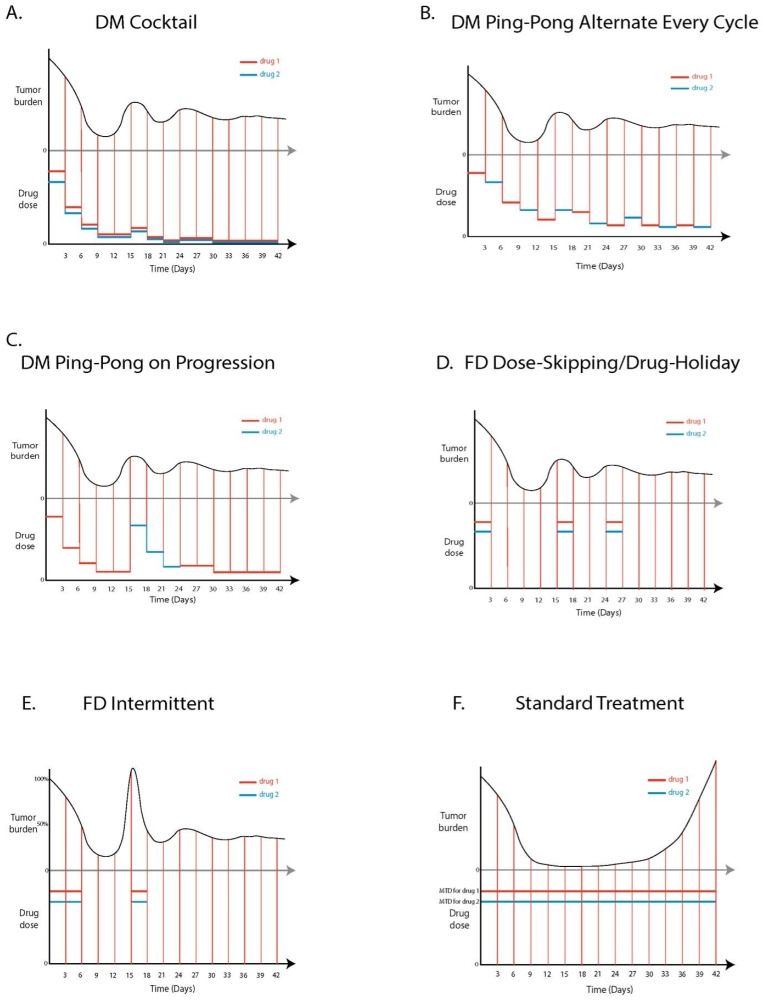
Two-drug adaptive therapy protocols, comparing variations of dose modulation (DM) and fixed dose (FD) adaptive therapy. Each panel shows on top, an example of how tumor burden might fluctuate over time, and below, how the dosing of the two drugs would be adjusted in response to the change in the tumor burden. Tumor burden is measured every 3 days (indicated with vertical lines). (**A**) DM Cocktail increases the dose of both drugs if the tumor is growing, and reduces the dose of both drugs if the tumor is shrinking. (**B**) DM Ping-Pong Alternate Every Cycle uses one drug at a time, but alternates drugs every 3 days, and adjusts the dose depending on how the tumor responded to the drug the last time it was applied. (**C**) DM Ping-Pong on Progression also uses one drug at a time, reducing the dose if the tumor is shrinking, but switching drugs if the tumor grows. (**D**) FD Dose-Skipping/Drug Holiday is similar to the AT-2 algorithm from [17], a fixed dose is applied every time the tumor grows, but the dose is skipped if the tumor remains stable or shrinks. (**E**) FD Intermittent is similar to the adaptive therapy prostate cancer trial from [18], where a fixed dose is applied until the tumor shrinks below 50% of its initial size. Dosing is restarted if the tumor grows above 100% of its original size. Tick marks on the tumor burden axis at 50% and 100% represent absolute values that trigger administering or withholding dosages of drugs for FD Intermittent. (**F**) Standard treatment applies both drugs at maximum tolerated dose (MTD).

MTD for either drug administered singly was 5 units. We assumed that due to increased toxicity of combining drugs, the maximum that could be applied was 3 units of each drug when they were used in combination. For fixed dose adaptive therapy protocols, we used a drug cocktail that was 75% of the MTD for each drug (0.75 of 3 units of each drug, which was 2.25 units of each drug [20]), however we did not observe a difference between 75% and MTD for fixed dose protocols. We investigated five different multi-drug adaptive therapy protocols and compared them to a standard treatment as follows (Figure 3).

##### Standard Treatment (ST)

Both drugs (drug 1 and drug 2) were administered at maximum tolerated dose (MTD) in a cocktail formulation once every 24 h for the entire duration of the simulation (Figure 3F).

##### DM Cocktail Tandem

Treatment started at MTD for both drugs, and dosages of both drugs were adjusted simultaneously according to the dose modulation adaptive therapy protocol, parameterized by Delta Tumor and Delta Dose (Figure 3A). This was equivalent to the standard dose modulation adaptive therapy protocol (AT-1) from previous experiments [19,20], but using two drugs in tandem, as if they were one.

##### DM Ping-Pong Alternate Every Cycle

Treatment started with drug 1 at MTD followed by drug 2 at MTD during the subsequent cycle. Drugs were always switched every cycle and dosages of each drug were adjusted (Delta Dose) based on the response of the tumor (Delta Tumor) the last time the same drug was administered (Figure 3B).

##### DM Ping-Pong on Progression

As in the standard dose modulation protocol with a single drug, we decreased the dose by Delta Dose when the tumor shrank by at least Delta Tumor. However, if the tumor grew by more than Delta Tumor, instead of increasing the dose of the current drug, we switched to the other drug. Any time we resumed the use of a drug that was used previously, we restarted treatment with a dose that was Delta Dose higher than the last time that drug was used (because the tumor grew the last time that drug was used at the prior concentration). Initially, each drug was started at MTD. Therefore, as long as the tumor was stable or shrinking, we continued using the current drug (Figure 3C).

##### FD Dose-Skipping/Drug Holiday

Drugs were administered in a cocktail formulation at a fixed dose that was set at 75% of the MTD. If the tumor grew by more than Delta Tumor since its last measurement, or if the tumor burden exceeded its previous maximum size, the drug was applied. Otherwise, the dose was skipped (Figure 3D). This was the AT-2 protocol from [20], except we used two drugs in combination.

##### FD Intermittent

Treatment started at 75% of the MTD using a cocktail formulation. Drug was administered once every 24 h. Treatment stopped any time the tumor burden fell by at least 50% of the value at which treatment was initiated. Treatment restarted if the tumor burden ever grew by at least 100% of the value at which treatment was initiated (Figure 3E). This was the protocol that was used for abiraterone in the prostate cancer clinical trial [21], except that we started and stopped two drugs in combination, rather than one.

## 3. Results

### 3.1. Dose Modulation Adaptive Therapy Protocols with Two Drugs Leads to Increased Time to Progression (TTP) Relative to Standard Treatment (ST) at Maximum Tolerated Dose

**Figure 4:**
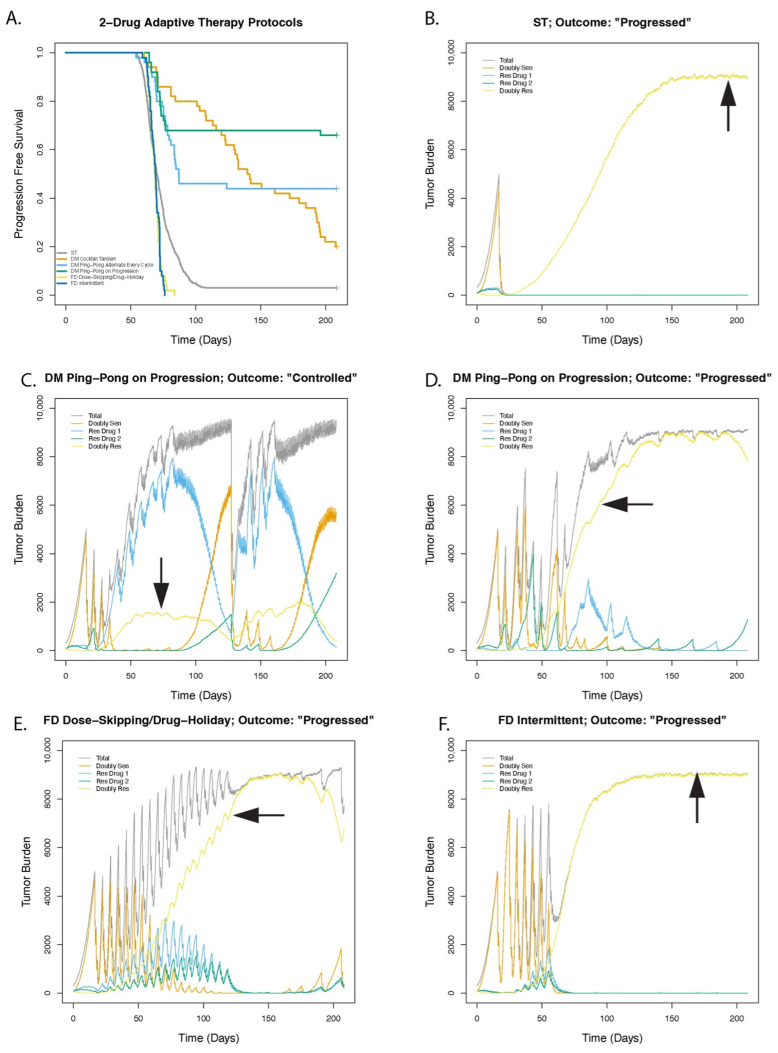
Two-drug therapies, comparing standard of care standard treatment (ST) versus variations of dose modulation (DM) and fixed dose (FD) adaptive therapy. Tumor burden was measured every 3 days. ST applied the maximum tolerated dose at each measurement. (**A**) Survival curves for DM adaptive therapy protocols (DM Cocktail Tandem, DM Ping-Pong Alternate Every Cycle, and DM Ping-Pong on Progression) and FD adaptive therapy protocols (FD Dose-Skipping/Drug Holiday and FD Intermittent) compared with ST. The dose modulation protocols uniformly worked better than the other protocols. (**B**) Cell population dynamics for a tumor treated with the ST protocol (continuous MTD). Therapy started at about day 20 when the tumor reached 5000 cells. The results clearly show the effects of competitive release, leading to rapid progression. (**C**) Cell population dynamics for a tumor treated with the DM Ping-Pong on Progression protocol, controlling the doubly resistant cells. There was a dip in tumor burden at about 125 days owing to switching from a low dose of drug 1 to a high dose of drug 2. (**D**) Cell population dynamics for a tumor treated with the DM Ping-Pong on Progression protocol resulting in the less frequent outcome of progression. At around day 70, therapy killed almost all the sensitive and singly resistant cells, leaving insufficient cells to keep the doubly resistant cells in check. (**E**) Cell population dynamics for a tumor treated with FD Dose-Skipping/Drug Holiday resulting in rapid progression. (**F**) Cell population dynamics for a tumor treated with the FD Intermittent protocol resulting in progression. Populations of the doubly resistant cells (in yellow) are indicated by arrows. In addition, the total tumor burden (gray), the number of cells sensitive to both drugs (Doubly Sen, in orange), the number of cells resistant to drug 1 but sensitive to drug 2 (Res Drug 1, in sky blue), and the number of cells resistant to drug 2 but sensitive to drug 1 (Res Drug 2, in bluish green), are shown.

Relative to standard of care treatment (ST) at maximum tolerated dose, we observed improved TTP with DM protocols (DM Cocktail: HR = 0.25 [0.18–0.35], *p* < 0.001; DM Ping-Pong Alternate: HR = 0.26 [0.18–0.38], *p* < 0.001; and DM Ping-Pong on Progression: HR = 0.13 [0.08–0.22], *p* < 0.001), but not the FD protocols, which actually performed worse than ST (FD Intermittent: HR = 1.67 [1.25–2.24], *p* < 0.001; FD Dose-Skipping/Drug Holiday: HR = 1.65 [1.23–2.21], *p* < 0.001), (Figure 4A and Supplementary Table S1). The average total amount of drug used in the adaptive therapy protocols was less than the standard treatment (DM Cocktail: 52.1% (drug 1) and 52.1% (drug 2); DM Ping-Pong Alternate: 32.6% (drug 1) and 66.5% (drug 2); DM Ping-Pong on Progression: 34.0% (drug1) and 39.8% (drug 2); FD Intermittent: 66.2% (drug 1) and 66.2% (drug 2); and FD Dose-Skipping/Drug Holiday: 35.9% (drug 1) and 35.9% (drug 2) of ST). Figure 4B–F shows the population dynamics of the different cell types over time for example runs. The number of doubly resistant cells (shown in red) progressively increased for ST (Figure 4B). FD Dose-Skipping/Drug Holiday and FD Intermittent (Figure 4E,F) also led to treatment failure. For treatment with DM Ping-Pong on Progression, Figure 4C shows an example run in which the number of doubly resistant cells were kept in check, while Figure 4D depicts the less common case where dose modulation failed. Preventing reverse mutations, which may better model genetic resistance mutations, did not significantly change the results (Supplemental Figure S2 and Table S8).

### 3.2. Greater Fitness Costs for Resistant Cells Increases the TTP for Adaptive Therapy

**Figure 5:**
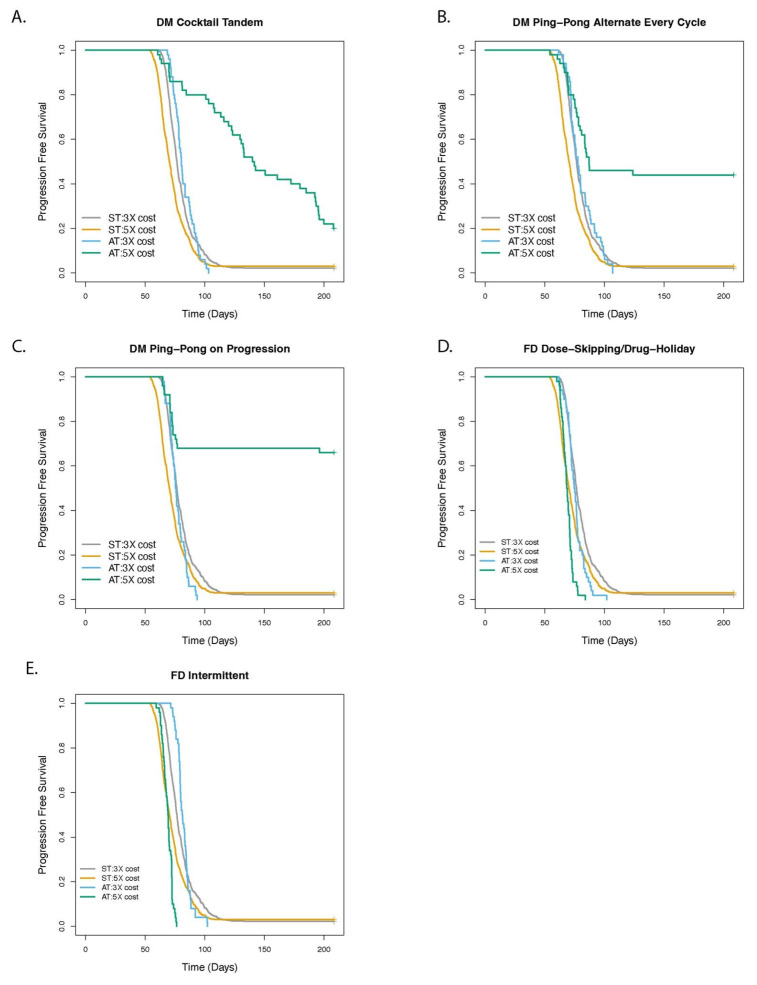
Role of fitness cost in determining the outcome of adaptive therapy with 2 drugs. The panels show the comparison of adaptive therapy (AT) versus standard treatment (ST) as fitness cost for resistance is varied for (**A**) DM Cocktail Tandem, (**B**) DM Ping-Pong Alternate Every Cycle, (**C**) DM Ping-Pong on Progression, (**D**) FD Dose-Skipping/Drug-Holiday, (**E**) FD Intermittent. 5× fitness cost is the default, with the division rate of doubly sensitive cells being 0.06/h, doubly resistant cells being 0.02/h, and that of the singly resistant cells being 0.04/h, while the death rate of all cell types was 0.01/h, translating to a net growth rate of the doubly sensitive cells at 5 times (5×) that of the doubly resistant cells. We compared this to a 3× fitness cost, with the division rate of doubly sensitive cells being 0.04/h, doubly resistant cells being 0.02/h, and singly resistant cells being 0.03/h, while the death rate of all cell types was 0.01/h, translating to a net growth rate of the doubly sensitive cells at 3 times (3×) that of the doubly resistant cells.

We explored the impact of the fitness cost parameter on TTP for treatment with ST and the adaptive therapy protocols (Figure 5 and Supplementary Table S2). Treatment with every dose modulation protocol increased TTP relative to ST (DM Cocktail: HR = 0.25 [0.18–0.35], *p* < 0.001; DM Ping-Pong Alternate: HR = 0.26 [0.18–0.38], *p* < 0.001; and DM Ping-Pong on Progression: HR = 0.13 [0.08–0.22], *p* < 0.001) when the fitness penalty incurred by the resistant cells was 5X (meaning that the net growth rate of the doubly sensitive cells was five times that of the doubly resistant cells, and the net growth rate of the singly resistant cells was three times that of the doubly resistant cells). When the fitness penalty incurred by the doubly resistant cells was 3X (and singly resistant cells had twice the growth rate of the doubly resistant cells), treatment with the dose modulation protocols relative to ST results in TTP was either not significantly different (DM Cocktail: not significant [*p* = 0.297]; DM Ping-Pong Alternate: not significant [*p* = 0.706]) or was worse (DM Ping-Pong on Progression: HR = 1.34 [1.00–1.79], *p* = 0.0482). In contrast, the fixed dose protocols, FD Intermittent and FD Dose-Skipping/Drug Holiday, were either worse or not significantly different than ST, regardless of whether or not the fitness cost for the resistance cells was 3X (FD Intermittent: not significant [*p* = 0.344]; FD Dose-Skipping/Drug Holiday: HR = 1.47 [1.10–1.97], *p* = 0.00851) or 5X (FD Intermittent: HR = 1.67 [1.25–2.24], *p* < 0.001; FD Dose-Skipping/Drug Holiday: HR = 1.65 [1.23–2.21], *p* < 0.001).

### 3.3. Higher Levels of Cell Turnover Increases the Efficacy of Adaptive Therapy

**Figure 6:**
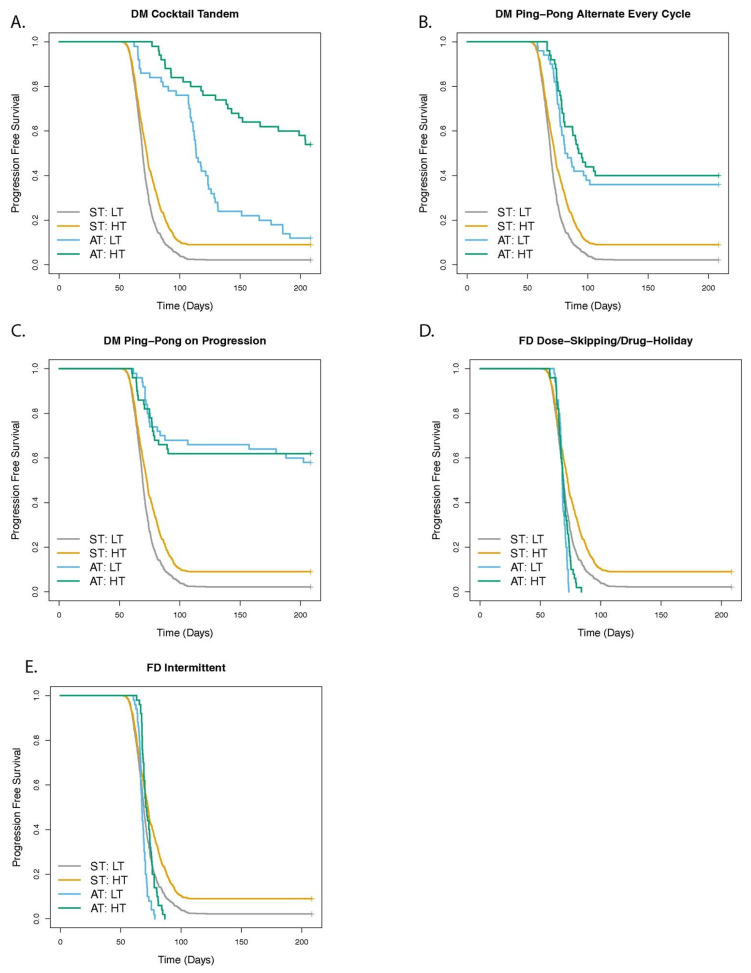
Effect of turnover on outcome of adaptive therapy with 2 drugs. The panels show the comparison of adaptive therapy (AT) versus standard treatment (ST) as turnover is varied while keeping the doubling time and net growth rate identical for (**A**) DM Cocktail Tandem, (**B**) DM Ping-Pong Alternate Every Cycle, (**C**) DM Ping-Pong on Progression, (**D**) FD Dose-Skipping/Drug-Holiday, (**E**) FD Intermittent. For low turnover (LT) conditions, the death rate was half of the default, at 0.005/h for all cell types, while for high turnover (HT) conditions, the death rate was twice the default at 0.02/h for all cell types. Division rates were set for each cell type to keep the fitness differences (net growth rates) the same as the default conditions. The dose modulation protocols worked well regardless of the amount of turnover. High cell turnover led to statistically significantly improved TTP in ST, DM Cocktail Tandem, and though the effect size was small, in both FD protocols.

For any given net growth rate, there can be more or less cell turnover generating that rate (determined by the cell death and division probabilities). We explored how the degree of cell turnover impacted adaptive therapy protocols (Figure 6). We kept the net growth rates of the different cell types the same as our default parameters (Table 2), but defined a low turnover condition with a cell death rate of 0.005/h for all cell types (half of the default value of 0.01/h) and division rates of doubly sensitive cells at 0.055/h, singly resistant cells at 0.035/h, and doubly resistant cells at 0.015/h. The high turnover condition had a death rate of 0.02/h for all cell types (twice the default), division rates of 0.07/h for the doubly sensitive cells, 0.05/h for the singly resistant cells, and 0.03/h for the doubly resistant cells. The doubling times in Table 2 were within the range of observed human cell division times, namely, from lymphocytes that can divide in less than 10 h [62,63,64], to common cancer cell lines in culture that range from 17 to 80 h doubling times [65].

Treatment with every dose modulation protocol improved time to progression, relative to ST, when cell turnover was low (DM Cocktail: HR = 0.25 [0.18–0.35, *p* < 0.001; DM Ping-Pong Alternate: HR = 0.28 [0.19–0.40], *p* < 0.001; DM Ping-Pong on Progression: HR = 0.14 [0.09–0.22], *p* < 0.001), and also when cell turnover was high (DM Cocktail: HR = 0.20 [0.13–0.30], *p* < 0.001; DM Ping-Pong Alternate: HR = 0.35 [0.24–0.51], *p* < 0.001; DM Ping-Pong on Progression: HR = 0.22 [0.14–0.34], *p* < 0.001).

DM Cocktail Tandem protocol worked particularly well where cell turnover was high, versus when it was low (HR = 0.29 [0.17–0.49], *p* < 0.001), whereas the amount of turnover had no significant effect on the success of the other two dose modulation protocols (DM Ping-Pong Alternate: *p* = 0.424; DM Ping-Pong on Progression: *p* = 0.834). ST also worked better when there were high levels of turnover in the tumor (HR = 0.68 [0.61–0.76], *p* < 0.001), relative to low turnover, as did the fixed dose adaptive therapy protocols (FD Intermittent: 0.39 [0.26–0.60], *p* < 0.001; FD Dose-Skipping/Drug Holiday: HR = 0.61 [0.39–0.93], *p* = 0.0234), but again, the fixed dose adaptive therapy protocols performed worse than ST, when cell turnover was high (FD Intermittent: HR = 1.44 [1.07–1.92], *p* = 0.0151; FD Dose-Skipping/Drug Holiday: HR = 1.90 [1.42–2.55], *p* < 0.001), in addition to when cell turnover was low (FD Intermittent: HR = 1.58 [1.18–2.11], *p* = 0.00213; FD Dose-Skipping/Drug Holiday: HR = 1.60 [1.19–2.14], *p* = 0.00175).

Tumor doubling times were typically much slower than cell culture doubling times. To investigate this, we tested reducing all of the division rates and death rates by an order of magnitude. This had the effect of exposing the cells to an order of magnitude more drug before they could divide. None of the simulated tumors in any of the protocols progressed within the 200 days, used in our other experiments, but eventually they all progressed (Supplementary Figure S3). Under these much slower kinetics, most of the adaptive therapy protocols were not statistically significantly better than standard therapy, in fact, DM Cocktail was worse. Only FD Dose-Skipping/Drug Holiday performed better than standard therapy (Supplementary Table S9).

### 3.4. Cell Replacement Increases the TTP with Adaptive Therapy

**Figure 7:**
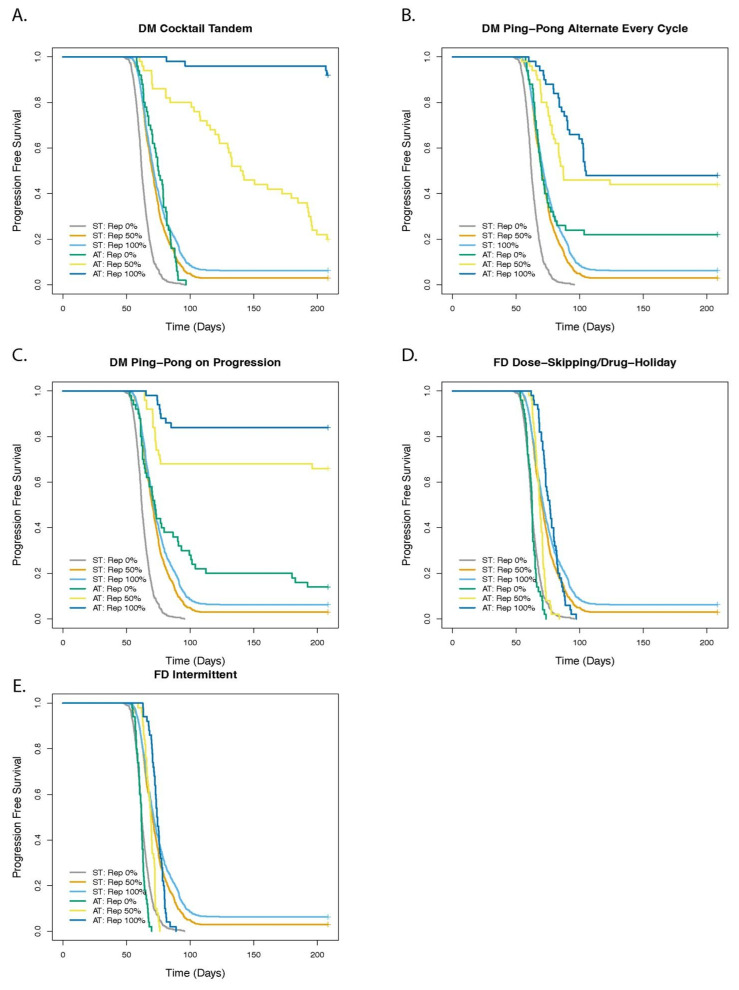
Effect of replacement on outcome of adaptive therapy with 2 drugs. The panels show the comparison of adaptive therapy (AT) versus standard treatment (ST) as the replacement parameter is varied for (**A**) DM Cocktail Tandem, (**B**) DM Ping-Pong Alternate Every Cycle, (**C**) DM Ping-Pong on Progression, (**D**) FD Dose-Skipping/Drug-Holiday, (**E**) FD Intermittent. The replacement parameter determined the probability that a dividing cell with no empty neighbors could replace a neighbor. We tested the two extremes in which a cell can always replace a neighbor (Rep 100%), representing direct cell competition, cancer cell cannibalism, or cell death due to crowding. We represented complete contact inhibition when a cell can never replace a neighbor (Rep 0%). We also tested an intermediate value (our default) in which a dividing cell can replace its neighbor 50% of the time (Rep 50%), representing some cell death due to crowding and other forms of competition, but also some degree of contact inhibition.

We investigated the role of spatial structure and the ability of cells to replace their neighbors (Figure 7 and Supplementary Table S4). If cells could not replace their neighbors, then they had to wait for a neighbor to die before they could reproduce in a crowded area of the tumor. Treatment worked better the more readily cells could replace their neighbors for all protocols (*p* < 0.01 and HR ≤ 0.83), with only two exceptions: for DM Ping-Pong Alternate Every Cycle, there was no significant difference between 50% and 100% replacement (*p* = 0.275), and for DM Ping-Pong on Progression, that difference was only modestly significant, though still with a large effect size (HR = 0.40 [0.17–0.92], *p* = 0.031). Dose modulation AT worked better than ST (*p* < 0.001 and HR ≤ 0.32 in all cases) and there was little difference between ST and FD protocols (see Supplementary Table S4 for hazard ratios, *p*-values, and Cox regression *p*-values for all comparisons). For the DM Cocktail protocol, the degree of replacement among the cells had a particularly strong effect (Figure 7A), with the hazard ratio = 0.08 [0.04–0.16], *p* < 0.001 when comparing 0% vs. 50% replacement, and the hazard ratio = 0.05 [0.02–0.15], *p* < 0.001 when comparing 50% vs. 100% replacement.

### 3.5. Adaptive Therapy Works Better If Smaller Changes in the Tumor Burden Trigger a Change in Dose

**Figure 8:**
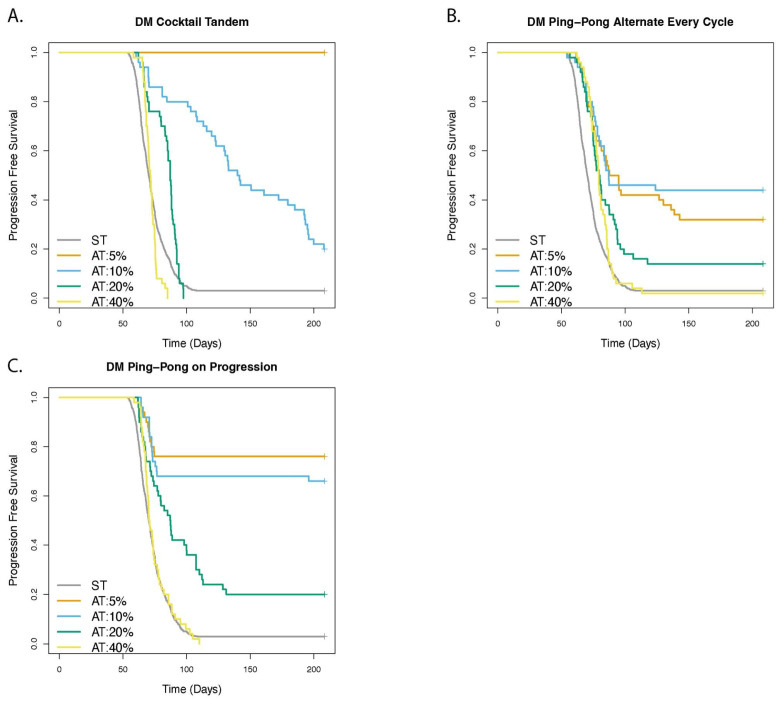
Delta Tumor is an important parameter determining outcome of dose modulation (DM) adaptive therapy. The panels show the comparison of adaptive therapy (AT) versus standard treatment (ST) as the Delta Tumor parameter is varied for (**A**) DM Cocktail Tandem, (**B**) DM Ping-Pong Alternate Every Cycle, (**C**) DM Ping-Pong on Progression. For the dose modulation (DM) protocols, Delta Tumor is the tumor measurement parameter specifying a relative value by which the tumor burden must change, compared with the last time it was measured, in order to trigger a change in drug dosage.

We investigated the role of the Delta Tumor parameter that determined how much the tumor burden must change before we changed the dose in the dose modulation protocols, or skipped a dose in the FD protocols (Figure 8, Supplementary Figure S1, and Supplementary Table S5). The dose modulation protocols were better than ST for all values of Delta Tumor (HR < 0.57 and *p* < 0.001). Treatment with DM Cocktail and Delta Tumor = 5%, achieved 100% survival and we observed no cases of progression. However, increasing the Delta Tumor value, that is, Delta Tumor = 40%, resulted in a TTP that was either significantly better than (DM Ping-Pong Alternate Every Cycle: HR = 0.67 [0.50–0.90], *p* = 0.00795), or not significantly different (DM Cocktail: *p* = 0.166; DM Ping-Pong on Progression: *p* = 0.545) from ST.

For the dose modulation protocols, TTP was progressively worse (HR ≥ 1.97 and *p* ≤ 0.00571) as we increased the value of the Delta Tumor from 5 to 10 to 20%, with two exceptions where these effects were not significant: increasing Delta Tumor from 5% to 10% for DM Ping-Pong Alternate (*p* = 0.397) and DM Ping-Pong on Progression (*p* = 0.356). As there was no recorded case of progression for DM Cocktail with Delta Tumor = 5% we were unable to calculate a hazard ratio but the TTP was clearly better for Delta Tumor = 5% versus 10% (Figure 8A, Chi Sq. *p* < 0.001).

Time to progression for treatment with the fixed dose protocols (FD Dose-Skipping/Drug Holiday and FD Intermittent) was either not significantly different or worse than treatment with ST (Supplementary Figure S1A,B, Supplementary Table S5). For FD Dose-Skipping/Drug Holiday, increasing Delta Tumor from 5% to 10% (*p* = 0.484), 10% to 20% (*p* = 0.254), or 20% to 40% (*p* = 0.51), did not result in any significant difference in TTP. For FD Intermittent, we stopped treatment when the tumor burden fell below the given percentage value relative to the initial tumor burden, not the last measure of tumor burden. Therefore, we investigated stopping treatment when the tumor shrank to 50% of the initial baseline for treatment initiation (the default), in addition to 80%, 90%, or 95% of the initial baseline for treatment initiation (Supplementary Figure S1B), though changing this parameter had no significant effects on TTP (50% vs. 80 [*p* = 0.0767], 80% vs. 90% [*p* = 0.13], and 90% vs. 95% [*p* = 0.432]).

### 3.6. For Dose Modulation Regimens, the Amount by Which the Drug Dose Is Changed (Delta Dose) Has Little Effect on the Success of Adaptive Therapy

**Figure 9:**
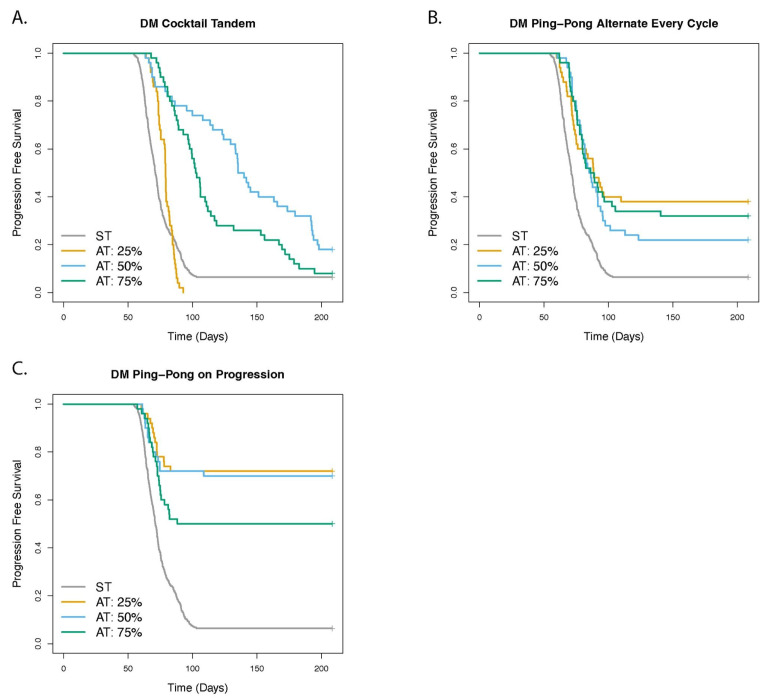
Role of the Delta Dose parameter for dose modulation (DM) adaptive therapy protocols. The panels show the comparison of adaptive therapy (AT) versus standard treatment (ST) as the Delta Dose parameter is varied for (**A**) DM Cocktail Tandem, (**B**) DM Ping-Pong Alternate Every Cycle, (**C**) DM Ping-Pong on Progression. Delta Dose is the percentage by which the drug dose is changed (increased or decreased) relative to the last time the same drug was administered. Default value is 50% for both drugs.

For the dose modulation protocols, we investigated the effect of a range of Delta Dose values (25%, 50%, or 75%) which determined how much we changed the dose relative to the last application of the drug, when the tumor burden changed by Delta Tumor (Figure 9 and Supplementary Table S6). In every case the TTP was improved relative to ST, that is, for Delta Dose = 25% (DM Ping-Pong Alternate: HR = 0.37 [0.26–0.53], *p* < 0.001; DM Ping-Pong on Progression: HR = 0.13 [0.08–0.23], *p* < 0.001), Delta Dose = 50% (DM Cocktail: HR = 0.34 [0.24–0.47], *p* < 0.001; DM Ping-Pong Alternate: HR = 0.46 [0.33–0.64], *p* < 0.001; DM Ping-Pong on Progression: HR = 0.15 [0.09–0.25], *p* < 0.001), and Delta Dose = 75% (DM Cocktail: HR = 0.40 [0.29–0.54], *p* < 0.001; DM Ping-Pong Alternate: HR = 0.39 [0.27–0.55], *p* < 0.001; DM Ping-Pong on Progression: HR = 0.29 [0.20–0.44], *p* < 0.001), with the exception of DM Cocktail, where dose adjustment by Delta Dose = 25% had no significant effect relative to treatment with ST (*p* = 0.355). For both DM Ping-Pong Alternate and DM Ping-Pong on Progression, increasing the value of Delta Dose from 25 to 50% (DM Ping-Pong Alternate: *p* = 0.306; DM Ping-Pong on Progression: *p* = 0.754), or 50 to 75% (DM Ping-Pong Alternate: *p* = 0.451; DM Ping-Pong on Progression: *p* = 0.0795), did not have a significant effect on TTP. However, for DM Cocktail, changing the dose by 50% was better than changing it by either 25% or 75% (increasing Delta Tumor from 25 to 50%: HR = 0.09 [0.04–0.18], *p* < 0.001; increasing Delta Tumor from 50 to 75%: HR = 1.74 [1.13–2.67], *p* = 0.0112). These results suggest that the success of adaptive therapy is not very sensitive to change in the Delta Dose parameter, as long as it is kept above a certain threshold for DM Cocktail, where it pays to not be too conservative with this parameter.

### 3.7. Dose Modulation Adaptive Therapy Works Best When Frequent Treatment Vacations Are Allowed

**Figure 10:**
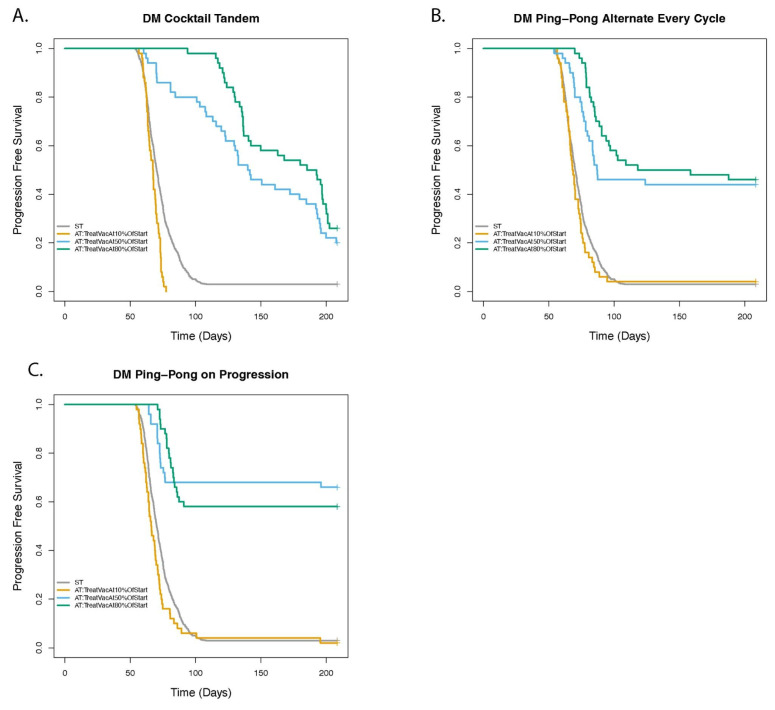
The effect of stopping treatment when the tumor burden falls below some threshold. The panels show the comparison of adaptive therapy (AT) versus standard treatment (ST) as the treatment vacation parameter is varied for (**A**) DM Cocktail Tandem, (**B**) DM Ping-Pong Alternate Every Cycle, (**C**) DM Ping-Pong on Progression. In the DM protocols, doses are adjusted as the tumor burden changes. However, a treatment vacation is triggered when the tumor burden falls below a certain threshold, resulting in no drug being administered for the treatment cycle, and treatment is resumed if the tumor regrows above that threshold. In the clinic this is often performed when the tumor is no longer detectable. Default value of the treatment vacation parameter for the DM protocols was 50% of the value at which therapy was initiated (TreatVacAt50%OfStart), that is, 25% of the carrying capacity. No statistically significant improvement in TTP was observed between 50% and 80% but 10% vs. 50% was statistically significant for all DM protocols, suggesting that we should stop dosing altogether as soon as is feasible, and not wait for the tumor to disappear.

For the dose modulation protocols, we investigated how the stopping conditions for dosing (triggering a treatment vacation) affects TTP (Figure 10 and Supplementary Table S7). We note that, for the dose modulation protocols, relative to ST, it was better to stop dosing when we could still detect the tumor, e.g., at a threshold of 50% (DM Cocktail: HR = 0.25 [0.18–0.35], *p* < 0.001; DM Ping-Pong Alternate: HR = 0.26 [0.18–0.38], *p* < 0.001; DM Ping-Pong on Progression: HR = 0.13 [0.08–0.22], *p* < 0.001), or 80% (DM Cocktail: HR = 0.19 [0.13–0.26], *p* < 0.001; DM Ping-Pong Alternate: HR = 0.19 [0.13–0.28], *p* < 0.001; DM Ping-Pong on Progression: HR = 0.16 [0.11–0.25], *p* < 0.001) of the initial tumor burden. This was in contrast to waiting until the tumor had shrunk to very low or undetectable levels (as is traditional in oncology), here represented as 10% of the initial tumor burden, in which case the TTP was either worse (DM Cocktail: HR = 1.95 [1.45–2.61], *p* < 0.001; DM Ping-Pong on Progression: HR = 1.38 [1.03–1.84], *p* = 0.0311) or not significantly different than ST (DM Ping-Pong Alternate: *p* = 0.277). TTP was improved for every DM protocol when we moved from a 10% to a 50% threshold of the initial tumor burden for stopping treatment (DM Cocktail: HR = 0.05 [0.02–0.11], *p* < 0.001; DM Ping-Pong Alternate: HR = 0.25 [0.15–0.40], *p* < 0.001; DM Ping-Pong on Progression: HR = 0.13 [0.07–0.24], *p* < 0.001). However, there was no significant difference in the results comparing a 50% versus 80% threshold (DM Cocktail: *p* = 0.0966; DM Ping-Pong Alternate: *p* = 0.31; DM Ping-Pong on Progression: *p* = 0.76). Note that the dose modulation protocols did not work well when waiting for the tumor to shrink below 10% of its initial size before stopping dosing. This implies that these treatment vacations are a crucial aspect of the dose modulation protocols.

## 4. Discussion

Design of multi-drug adaptive therapy protocols is challenging, as the number of parameters and potential protocols increases exponentially with each additional drug. However, drug combinations may afford more opportunities for better tumor control, relative to any single drug. Here, we tackled the challenge of designing multi-drug adaptive therapy protocols by investigating the simplest case of multi-drug adaptive therapy, that is, treatment with two drugs. Questions abound as to whether the treatment protocol should be sequential or concomitant [23]. We note that the only adaptive therapy clinical trial published to date used a fixed dose of a single drug (combined with a backbone drug held on continuously) and the published mouse experiments also used a single drug [19,20]. We simulated five different multi-drug adaptive therapy protocols with two drugs, and a standard-of-care standard treatment (ST) at maximum tolerated dose, under various scenarios of tumor dynamics, with the goal of finding the most promising treatment protocols.

We showed that, relative to standard of care standard treatment (ST) at maximum tolerated dose, for the default values of the parameters tested, treatment with the DM protocols increased time to progression, but the FD protocols were worse than ST (Figure 4A and Supplementary Table S1). These results were consistent with preclinical adaptive therapy experiments in mice with breast cancer, where mice treated with a single drug regimen of paclitaxel, as per the dose modulation adaptive therapy (AT-1) algorithm, improved TTP in mice, while treatment as per the FD Dose-Skipping (AT-2) algorithm did not, and in fact appeared to be worse than standard therapy (STD) for the ER+ model [20]. In contrast, in the prostate clinical trial of adaptive therapy, the FD Intermittent protocol worked better than ST [21]. Importantly, this trial was in a relatively slow growing cancer (metastatic, castrate resistant, prostate cancer), a situation that we did not model. The dose modulation protocol, that our model suggests would have worked even better, has not been tested in any clinical trial.

Among the dose-modulation protocols, the ping-pong protocols often resulted in long term control of the tumors (Figure 4A), and used only 34.0% (drug 1) and 39.8% (drug 2) for DM Ping-Pong on Progression, 32.6% (drug 1) and 66.5% (drug 2) of the amount of drug used in standard treatment, whereas the tumors eventually progressed under the Cocktail Tandem protocol, in most cases (using only 52.1% (drug 1) and 52.1% (drug 2) of the drug used in standard treatment, over the same amount of time). This was probably because the ping-pong protocols only apply one drug at a time, so there was no direct selection for doubly resistant cells. This meant that we continued to control the tumor by switching drugs. In contrast, the Cocktail Tandem protocol always applied both drugs at the same time, favoring doubly resistant cells, and eventually leading to progression.

Adaptive therapy depends on the sensitive cells being able to out-compete the resistant cells, in the absence of drug, or in the presence of low doses of drug. In our model, this fitness differential had to be more than three-fold for the DM protocols, and a five-fold difference was not sufficient to make the FD protocols work (Figure 5 and Supplementary Table S2). It is possible that there is some level of fitness cost of resistance at which fixed dose protocols will work better than ST. It is worth noting that, we and others have shown that when using models that ignore the possibility of a return to sensitivity, then FD adaptive protocols with single drugs do work better than ST [17,42,43,66]. Whether resistance generally incurs a fitness cost, and what is the magnitude of the fitness penalty incurred by the resistant cells, are open questions. In one study, MCF7 cells resistant to Doxorubicin (MCF7Dox) had a doubling time of 60 h, while sensitive MCF7 (MCF7) cells had a doubling time of 40 h [17]. In Lotka–Volterra competition models of adaptive therapy, it is common practice to assign competition coefficients based on the assumption that resistance comes at a fitness cost [21,23]. While the fitness costs we modeled were high relative to what is typically observed in organismal evolution, they were not unreasonable given observations in clonal evolution of cancer [66]. Furthermore, Gatenby et al. presented calculations of the competition coefficients that best fit the data from the clinical trial [21] and found that the cells resistant to abiraterone had a 7X lower fitness than the sensitive cells. In contrast, even in our high fitness cost case where the doubly resistant cells incurred a 5X fitness cost, the singly resistant cells only incurred a 1.7X fitness cost compared with the sensitive cells.

The amount of cell turnover had little effect on the success of adaptive therapy, except in DM Cocktail Tandem which performed better at high levels of turnover (Figure 6 and Supplementary Table S3). In our previous modeling work, we underscored the importance of turnover in agent-based and mathematical models [43,49]. Increased turnover can improve adaptive therapy if there is a fitness cost of resistance, but higher fitness costs do not necessarily translate to an improved outcome in the absence of increased turnover [43]. However, this previous work only modeled a single drug and both the cost of resistance and cell turnover changed to fit the dynamics of the clinical data. This highlighted the possibility that these effects can occur simultaneously, and, in fact, trade off with one another. Here, we tried to disentangle the effects of fitness cost from turnover, by ensuring that the doubling time of each cell type was identical for the two scenarios (low and high turnover). In general, our model predicted that the fitness cost of resistance appeared more important than the degree of turnover (Figure 5 and Figure 6). We also found that the frequency of dosing relative to the cell doubling times could have dramatic effects on the success of all of the therapies (Supplementary Figure S3).

One of the most important parameters in the model was the probability that a dividing cell may replace its neighbor. In reality, it is not clear if a dividing cell can replace a neighbor. In fact, we do not know how the local density of a cancer cell affects its reproduction and survival. Stem cells are able to replace each other in a stem-cell niche [67,68], and there is evidence of cancer cell cannibalism (entosis) in which one cell kills its neighbor [56,57,69]. Because it is an open question regarding how cancer cells behave when there is no adjacent space available to divide, we introduced a replacement parameter in the model to represent a range of possibilities. We showed that it was possible to achieve improved TTP with dose modulation (DM) adaptive therapy protocols, relative to ST, regardless of whether a dividing cancer cell was able to replace a neighboring cell (Figure 7 and Supplementary Table S4), however dose modulation protocols worked substantially better the more that cells could replace their neighbors, which is a particular type of cell turnover.

Our results suggest that if we are to manage cancers based on their response to our therapies (i.e., Delta Tumor), we should be as sensitive as possible to changes in tumor burden, and adjust our therapy accordingly (Figure 8). In practice, this may be limited by the error in measurements of tumor burden (and the frequency with which we can measure it).

How much to change the dose when the tumor grows or shrinks (Delta Dose) is a key parameter for DM protocols. We found, for a wide range of Delta Dose values (25%, 50%, or 75%) that dose modulation protocols generally resulted in an improvement in TTP relative to ST (Figure 9 and Supplementary Table S6), and that there was often little difference in outcomes between the different values of Delta Dose, except in a few cases when Delta Dose was too low. These results also suggest we should not be too conservative about changing the dosages when the tumor burden changes. This is consistent with the results of a previous model using single-drug adaptive therapy, in which a dose modulation regimen with a Delta Tumor = 10% and Delta Dose = 50%, resulted in improved treatment outcome relative to continuous treatment at maximum tolerated dose (which we call standard treatment) [17]. In that study, the investigators compared Delta Tumor = 5% and Delta Dose = 25% versus Delta Tumor = 10% and Delta Dose = 50%, and found that the latter protocol worked better [17]. Our model suggested that Delta Tumor = 5% and Delta Dose = 50% would be even better.

Interestingly, we found that, for DM protocols to work, it was helpful for frequent treatment vacations to be incorporated in the protocols. Waiting too long to start a treatment vacation, e.g., waiting until the tumor burden fell to 10% of its initial level, resulted in a TTP that was either worse or not significantly different than ST (Figure 10 and Supplementary Table S7). We found good results when we stopped dosing if the tumor only shrank to 80% of its initial level. That would not even qualify as a partial response by RECIST 1.1 criteria [17,70]. This result is consistent with the current understanding that, the less drug dose we administer to a tumor, the less we select for therapeutic resistance.

Our work has some limitations. We did not explore the role of angiogenesis; three-dimensional tissue architecture composed of cancer cells, normal cells, immune cells and other stromal cells in the microenvironment of a tumor. We attempted to capture what happens in individual cross-sections of the tumor, which we assumed to be adequately perfused with capillaries, such that the drug diffusion and delivery was not limiting, and that the drug delivery was not limited by constraints imposed by tumor architecture and pressure inside the tumor. We also implicitly assumed an ability to transition freely between the four cell types, albeit driven by mutation or epigenetic modification [60,61], and while it does make the tumor more difficult to treat it also means that any cell that can divide can become doubly or singly resistant or sensitive with a single division. We have previously looked at the role of plasticity in adaptive therapy using a combination of in vitro/in vivo and mathematical modeling [71], in addition to clinical data [72]. In both cases, a key parameter was the switching rate between the cell types. Here, we used an unrealistically high mutation rate to compensate for an unrealistically low cell population size. We also used a computationally efficient abstraction of cell crowding dynamics and explored a range of parameters that modeled different possibilities for those dynamics. Future efforts might consider a range of different mutation rates, extend our model to three dimensions, model cells pushing neighbors aside, include additional hallmarks of cancer, and incorporate a more realistic representation of blood vessel dynamics, in addition to nutrient and drug delivery.

Importantly, while most models predict recurrence of the tumor due to resistant cells ultimately taking over in the tumor, our results suggest it is possible to maintain indefinite control over the tumor, lending support to the idea that it is possible to convert cancer from an acute disease that inevitably leads to death to a chronic disease that can be tolerated.

## 5. Conclusions

Our results suggested that, when combining drugs in adaptive therapies, dose modulation protocols were much better than fixed dose protocols, and ping-pong protocols were probably better than applying multiple drugs at the same time. Applying one drug at a time and switching when the tumor grew (DM Ping-Pong on Progression) worked best across many parameter variations, though adjusting the dose of both drugs at the same time could also work well (DM Cocktail Tandem), especially if we had a very sensitive measure of tumor burden (Figure 8A). One attractive feature of the ping-pong protocols is that only one drug is applied at a time, which may help to reduce toxicity and selection for multidrug resistance, compared with combination therapies. Furthermore, all adaptive therapy protocols reduce the amount of drug used over the same amount of time as standard therapy. However, if adaptive therapy is successful in controlling cancer indefinitely, the total amount of drug used will eventually exceed an MTD protocol that could not control a cancer. Dose modulation protocols are particularly effective when cell competition is more intense, and when dosing of the tumor is kept to a minimum. Furthermore, in our model, adaptive therapy worked better than standard treatment only when there was a relatively large fitness cost of resistance. This suggests that developing good biomarkers for measuring cell turnover, clonal competition for space, and the fitness cost of resistance, in addition to intra-tumor heterogeneity as a proxy for the likelihood that resistant cells are already present at diagnosis, will be important for distinguishing which cancers should be treated with adaptive therapy in the future. These predictions should be tested in pre-clinical models, and if supported there, further tested in clinical trials.

## Figures and Tables

**Table 1:** Parameter table with default values.

Parameter	Value
Cell division rate: doubly sensitive	0.06 per hour
Cell division rate: resistant to drug 1	0.04 per hour
Cell division rate: resistant to drug 2	0.04 per hour
Cell division rate: doubly resistant	0.02 per hour
Background death rate	0.01 per hour
Replacement probability	0.5
Delta Tumor	10%
Delta Dose	50%
Probability of death due to drug 1 potency (Ψ1)	0.04 per unit drug concentration
Probability of death due to drug 2 potency (Ψ2)	0.04 per unit drug concentration
Maximum tolerated dose (MTD)	5.0 units for a single drug. See Section 2.7.6 for MTD under combination therapies.
Minimum drug dose	0.5 units
Drug on time	1 h
Frequency of drug application	Once every 24 h
Check tumor burden	Every 3 days
Drug decay	10% per hour
Drug diffusion rate	2.0
Tumor size triggering treatment	Tumor burden is 50% or more of the carrying capacity
Mutation rate	10^−3^ per cell division
Measurement noise standard deviation (SD)	5 cells
Total grid size	100 by 100
Duration of simulation	5000 h
Stop dosing/initiate treatment vacation when (DM protocols only):	Tumor burden is less than or equal to 25% of carrying capacity

**Table 2:** Doubling time of the cell types.

Cell Types	Doubling Time
Doubly sensitive	13.86 h
Resistant to drug 1	23.1 h
Resistant to drug 2	23.1 h
Doubly resistant	69.3 h

## Data Availability

Source code and data are available from https://github.com/codeCan1/catreat, accessed on 20 May 2022.

## Supplementary Materials

The following are available online at https://www.mdpi.com/article/10.3390/cancers14112699/s1, Figure S1: Role of Delta Tumor parameter in determining outcome of fixed dose (FD) adaptive therapy protocols; Figures S2 and S3: Survival curves for dose modulation (DM) and fixed dose (FD) adaptive therapy protocols under various conditions; Tables S1–S9: Hazard ratios along with the 95% confidence intervals, *p*-values, and test for proportionality of hazards *p*-values for various dose modulation and fixed dose adaptive therapy protocols relative to standard treatment (ST), Fitness Cost Parameter, Turnover Parameter, Replacement Parameter, Delta Tumor Parameter, Delta Dose Parameter, Treatment Vacation Parameter, various dose modulation and fixed dose adaptive therapy protocols relative to standard treatment (ST) under conditions when cells can undergo forward mutations only but no reverse mutations; various dose modulation and fixed dose adaptive therapy protocols relative to standard treatment (ST) under conditions when the doubling time of each cell type is increased by one order of magnitude relative to the default values.
