# Supplementary material for: In Silico Investigations of Multi-Drug Adaptive Therapy Protocols
Source: Cancers (Basel). 2022 May 30;14(11):2699. doi: 10.3390/cancers14112699 (PMC9179496; doi:10.3390/cancers14112699)
Supplement: Supplementary file 1 [file cancers-14-02699-s001.zip › cancers-1691098-supplementary.pdf]

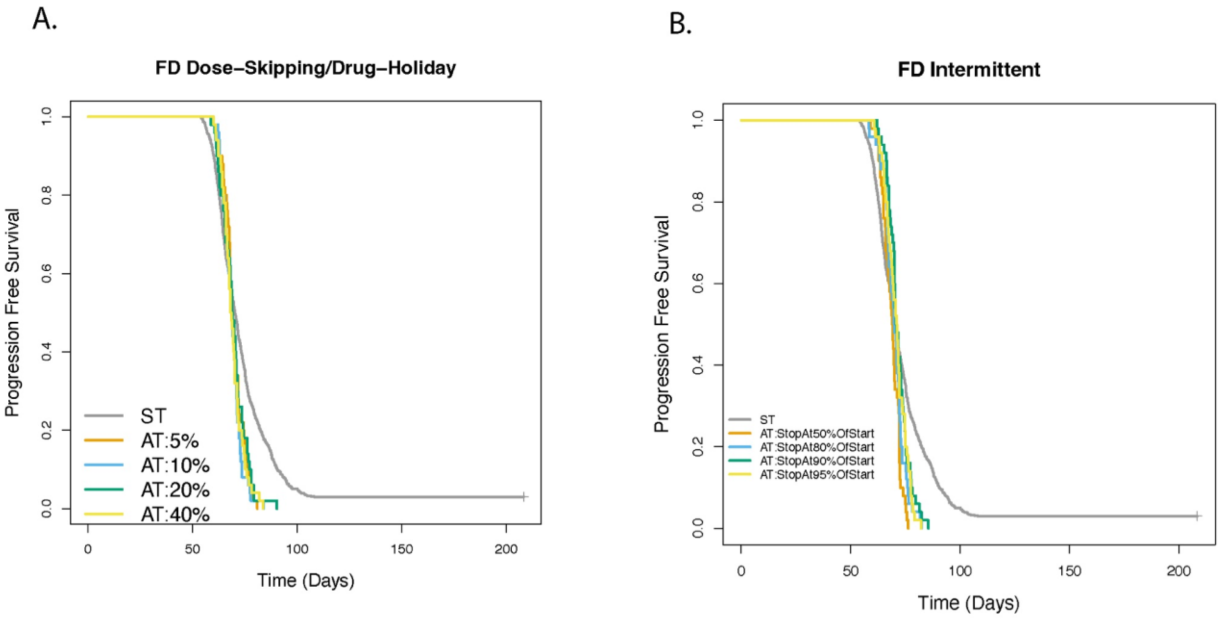

Figure S1. Role of Delta Tumor parameter in determining outcome of fixed-dose (FD) adaptive therapy protocols. For FD Dose-Skipping/Drug-Holiday, delta tumor is percentage change in tumor burden relative to the last measurement, such that a fixed dosage of the drugs is administered if the tumor burden exceeds the threshold, and treatment is skipped otherwise, default value being 10%. For FD Intermittent, Delta Tumor is the absolute value at which treatment is stopped relative to the baseline for treatment initiation, default being stopping treatment when tumor shrinks to 50% of the initial baseline for treatment initiation. The Fixed Dose (FD) protocols. **A.** FD Dose-Skipping/Drug-Holiday, **B.** FD intermittent are defined in the text.

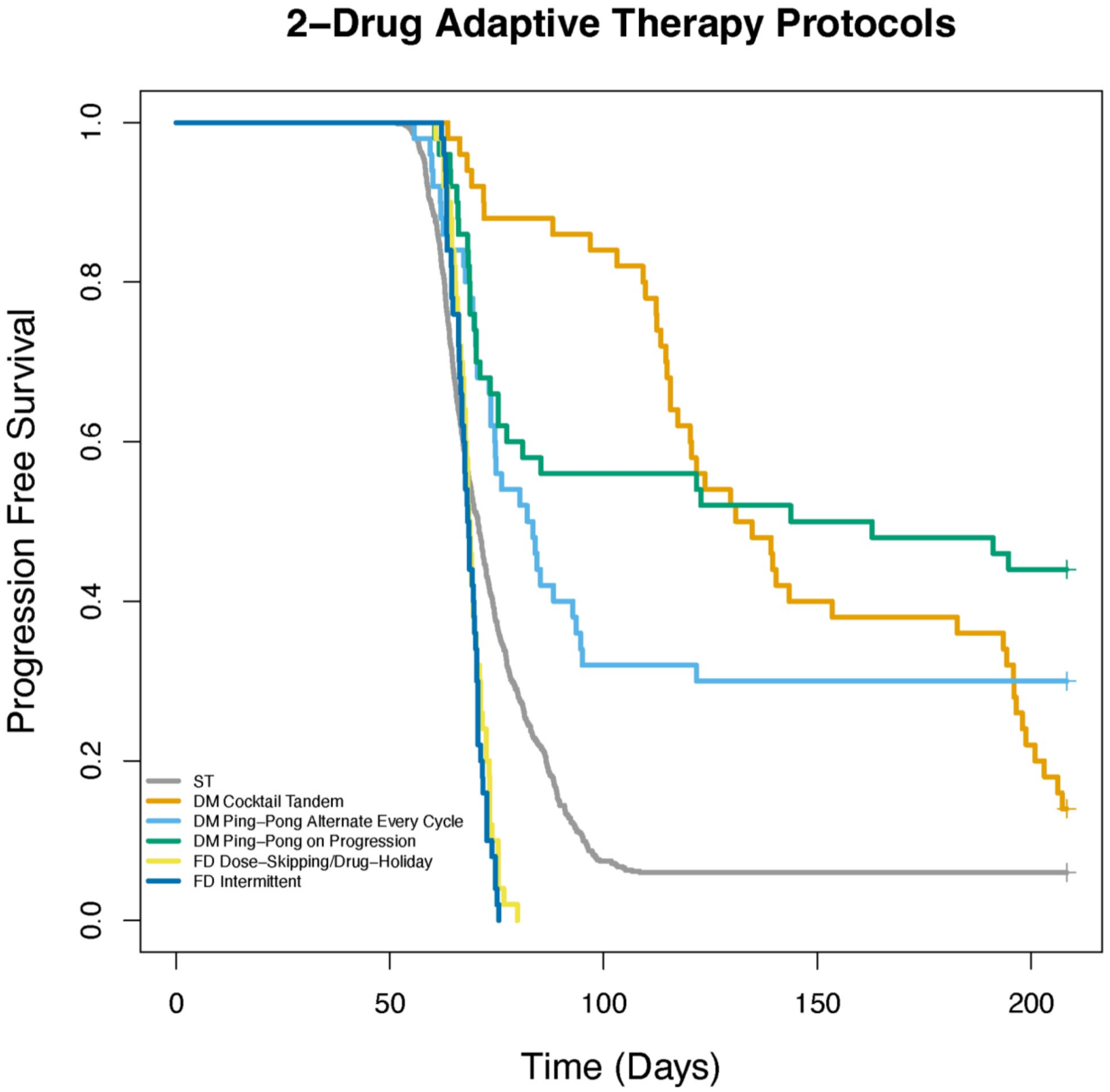

Figure S2. Survival curves when cells can undergo forward mutation to resistance phenotypes but no reverse mutations. Dose modulation (DM) adaptive therapy protocols still work better than fixed dose (FD) adaptive therapy protocols.

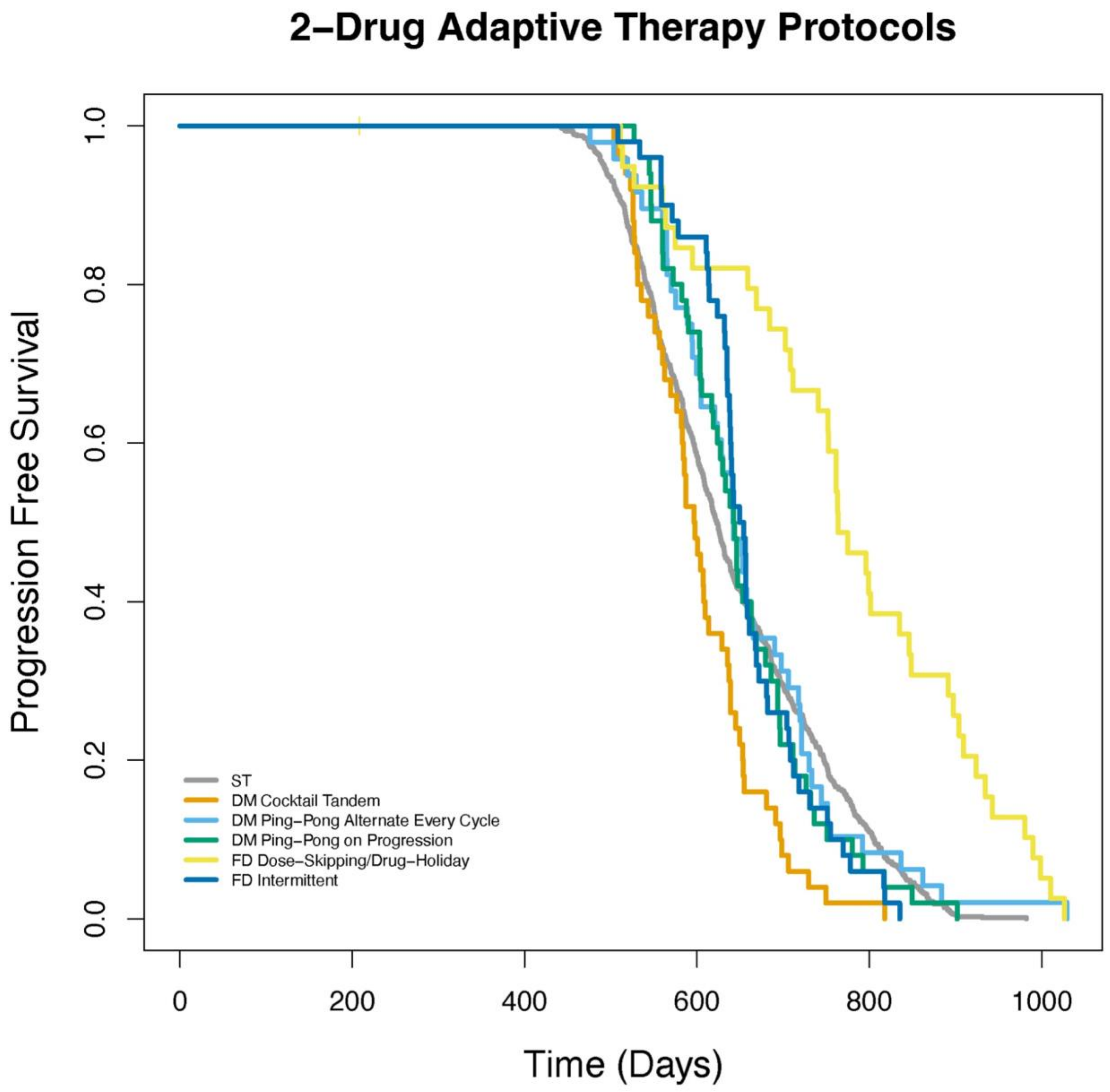

Figure S3. Survival curves when the doubling time of each cell type is increased by one order of magnitude relative to the default values, representing slow tumor doubling times. Note all therapies prevent progression for much longer than the 200 days we tested in previous experiments. Dose modulation (DM) adaptive therapy protocols and fixed dose (FD) adaptive therapy protocols are mostly equivalent to standard treatment (ST), though FD Dose-Skipping/Drug-Holiday is better, and DM Cocktail Tandem is worse.

Table S1: Hazard ratios along with the 95% confidence intervals (c.i.), *p*-values, and test for proportionality of hazards *p*-values for various dose-modulation and fixed-dose adaptive therapy protocols relative to standard treatment (ST).

Protocol	Hazard Ratio (95% c.i.) relative to standard treatment	*p* value	Test for proportionality of hazards *p* value
DM Cocktail	0.25 (0.18-0.35)	<0.001	0.0016
DM Ping-Pong Alternate Every Cycle	0.26 (0.18-0.38)	<0.001	0.66
DM Ping-Pong on Progression	0.13 (0.08-0.22)	<0.001	0.045
FD Intermittent	1.67 (1.25-2.24)	<0.001	1.6e-10
FD Dose-Skipping/Drug Holiday	1.65 (1.23-2.21)	<0.001	1.2e-7

Table S2. Hazard ratios along with the 95% confidence intervals (c.i.), *p*-values, and test for proportionality of hazards *p*-values for Fitness Cost Parameter. Abbreviations: n.s. is not significant, ST is standard treatment.

Comparison	Hazard Ratio (95% c.i.)	*p* value	Test for proportionality of hazards *p* value
ST (5X cost) relative to ST (3X cost)	1.47 (1.32-1.64)	<0.001	<2e-16
DM Cocktail (3X cost) relative to ST (3X cost)		n.s. (0.297)	
DM Cocktail (5X cost) relative to ST (5X cost)	0.25 (0.18-0.35)	<0.001	0.0016
DM Cocktail (5X cost) relative to DM Cocktail (3X cost)	0.09 (0.04-0.18)	<0.001	3.5e-8
DM Ping-Pong Alternate Every Cycle (3X cost) relative to ST (3X cost)		n.s. (0.706)	
DM Ping-Pong Alternate Every Cycle (5X cost) relative to ST (5X cost)	0.26 (0.18-0.38)	<0.001	0.66
DM Ping-Pong Alternate Every Cycle (5X cost) relative to DM Ping-Pong Alternate Every Dose (3X cost)	0.35 (0.21-0.56)	<0.001	3e-4
DM Ping-Pong on Progression (3X cost) relative to ST (3X cost)	1.34 (1.00-1.79)	0.0482	0.0052
DM Ping-Pong on Progression (5X cost) relative to ST (5X cost)	0.13 (0.08-0.22)	<0.001	0.045
DM Ping-Pong on Progression (5X cost) relative to DM Ping-Pong on Progression (3X cost)	0.17 (0.09-0.31)	<0.001	1.4e-8
FD Dose-Skipping/Drug Holiday (3X cost) relative to ST (3X cost)	1.47 (1.10-1.97)	0.00851	0.092
FD Dose-Skipping/Drug Holiday	1.65 (1.23-2.21)	<0.001	1.2e-7

(5X cost) relative to ST (5X cost)			
FD Dose-Skipping/Drug Holiday (5X cost) relative to FD Dose-Skipping (AT-2) (3X cost)	3.12 (2.03-4.80)	<0.001	0.23
FD Intermittent (3X cost) relative to ST (3X cost)		n.s. (0.344)	
FD Intermittent (5X cost) relative to ST (5X cost)	1.67 (1.25-2.24)	<0.001	1.6e-10
FD Intermittent (5X cost) relative to FD Intermittent (3X cost)	28.88 (12.73-65.54)	<0.001	0.17

Table S3. Hazard ratios along with the 95% confidence intervals (c.i.), *p*-values, and test for proportionality of hazards *p*-values for Turnover Parameter. Abbreviations: n.s. is not significant, LT is low turnover, HT is high turnover, ST is standard treatment.

Comparison	Hazard Ratio (95% c.i.)	*p* value	Test for proportionality of hazards *p* value
ST (HT) relative to ST (LT)	0.68 (0.61-0.76)	<0.001	0.1
DM Cocktail (LT) relative to ST (LT)	0.25 (0.18-0.35)	<0.001	0.0018
DM Cocktail (HT) relative to ST (HT)	0.20 (0.13-0.30)	<0.001	3.7e-6
DM Cocktail (HT) relative to DM Cocktail (LT)	0.29 (0.17-0.49)	<0.001	0.62
DM Ping-Pong Alternate Every Cycle (LT) relative to ST (LT)	0.27 (0.19-0.40)	<0.001	0.31
DM Ping-Pong Alternate Every Cycle (HT) relative to ST (HT)	0.35 (0.24-0.51)	<0.001	0.032
DM Ping-Pong Alternate Every Cycle (HT) relative to DM Ping-Pong Alternate Every Dose (LT)		n.s. (0.424)	
DM Ping-Pong on Progression (LT) relative to ST (LT)	0.14 (0.09-0.22)	<0.001	0.93
DM Ping-Pong on Progression (HT) relative to ST (HT)	0.22 (0.14-0.34)	<0.001	0.072
DM Ping-Pong on Progression (HT) relative to DM Ping-Pong on Progression (LT)		n.s. (0.834)	
FD Dose-Skipping/Drug Holiday (LT) relative to ST (LT)	1.60 (1.19-2.14)	0.00175	2.1e-10
FD Dose-Skipping/Drug Holiday (HT) relative to ST (HT)	1.90 (1.42-2.55)	<0.001	8.7e-7
FD Dose-Skipping/Drug Holiday (HT) relative to FD Dose-Skipping (AT-2) (LT)	0.61 (0.39-0.93)	0.0234	0.0019

FD Intermittent (LT) relative to ST (LT)	1.58 (1.18-2.11)	0.00213	4.5e-7
FD Intermittent (HT) relative to ST (HT)	1.44 (1.07-1.92)	0.0151	1.5e-10
FD Intermittent (HT) relative to FD Intermittent (LT)	0.39 (0.26-0.60)	<0.001	0.25

Table S4. Hazard ratios along with the 95% confidence intervals (c.i.), *p*-values, and test for proportionality of hazards *p*-values for Replacement Parameter. Abbreviations: n.s. is not significant, Rep is replacement, ST is standard treatment.

Comparison	Hazard Ratio (95% c.i.)	*p* value	Test for proportionality of hazards *p* value
ST (50% Rep) relative to ST (0% Rep)	0.36 (0.32-0.40)	<0.001	0.0016
ST (100% Rep) relative to ST (50% Rep)	0.83 (0.74-0.92)	<0.001	0.11
DM Cocktail (0% Rep) relative to ST (0% Rep)	0.32 (0.23-0.43)	<0.001	0.011
DM Cocktail (50% Rep) relative to ST (50% Rep)	0.25 (0.18-0.35)	<0.001	0.0016
DM Cocktail (100% Rep) relative to ST (100% Rep)	0.03 (0.01-0.08)	<0.001	0.097
DM Cocktail (50% Rep) relative to DM Cocktail (0% Rep)	0.08 (0.04-0.16)	<0.001	0.00033
DM Cocktail (100% Rep) relative to DM Cocktail (50% Rep)	0.05 (0.02-0.15)	<0.001	0.87
DM Ping-Pong Alternate Every Cycle (0% Rep) relative to ST (0% Rep)	0.28 (0.20-0.40)	<0.001	0.79
DM Ping-Pong Alternate Every Cycle (50% Rep) relative to ST (50% Rep)	0.26 (0.18-0.38)	<0.001	0.66
DM Ping-Pong Alternate Every Cycle (100% Rep) relative to ST (100% Rep)	0.23 (0.16-0.35)	<0.001	0.025
DM Ping-Pong Alternate Every Cycle (50% Rep) relative to DM Ping-Pong Alternate (0% Rep)	0.45 (0.27-0.73)	0.00128	0.028
DM Ping-Pong Alternate Every Cycle (100% Rep) relative to DM Ping-Pong Alternate (50% Rep)		n.s. (0.275)	

DM Ping-Pong on Progression (0% Rep) relative to ST (0% Rep)	0.24 (0.17-0.34)	<0.001	0.026
DM Ping-Pong on Progression (50% Rep) relative to ST (50% Rep)	0.13 (0.08-0.22)	<0.001	0.026
DM Ping-Pong on Progression (100% Rep) relative to ST (100% Rep)	0.07 (0.03-0.13)	<0.001	0.69
DM Ping-Pong on Progression (50% Rep) relative to DM Ping-Pong Progression (0% Rep)	0.23 (0.13-0.41)	<0.001	0.85
DM Ping-Pong on Progression (100% Rep) relative to DM Ping-Pong Progression (50% Rep)	0.40 (0.17-0.92)	0.0305	0.044
FD Dose-Skipping/Drug Holiday) (0% Rep) relative to ST (0% Rep)		n.s. (0.191)	
FD Dose-Skipping/Drug Holiday (50% Rep) relative to ST (50% Rep)	1.65 (1.23-2.21)	<0.001	1.2e-7
FD Dose-Skipping/Drug Holiday (100% Rep) relative to ST (100% Rep)		n.s. (0.938)	
FD Dose-Skipping/Drug Holiday (50% Rep) relative to FD Dose-Skipping (AT-2) (0% Rep)	0.31 (0.21-0.48)	<0.001	0.002
FD Dose-Skipping/Drug Holiday (100% Rep) relative to FD Dose-Skipping (AT-2) (50% Rep)	0.26 (0.17-0.41)	<0.001	0.47
FD Intermittent (0% Rep) relative to ST (0% Rep)	1.49 (1.12-1.99)	<0.00696	0.00043
FD Intermittent (50% Rep) relative to ST (50% Rep)	1.67 (1.25-2.24)	<0.001	1.6e-10
FD Intermittent (100% Rep) relative to ST (100% Rep)		n.s. (0.294)	

FD Intermittent (50% Rep) relative to FD Intermittent (0% Rep)	0.15 (0.09-0.25)	<0.001	0.089
FD Intermittent (100% Rep) relative to FD Intermittent (50% Rep)	0.25 (0.16-0.40)	<0.001	0.96

Table S5. Hazard ratios along with the 95% confidence intervals (c.i.), *p*-values, and test for proportionality of hazards *p*-values for Delta Tumor Parameter. Abbreviations: n.s. is not significant, ST is standard treatment.

Comparison	Hazard Ratio (95% c.i.)	*p* value	Test for proportionality of hazards *p* value
DM Cocktail (Delta Tumor 5%) relative to ST		n.s. (0.982)	
DM Cocktail (Delta Tumor 10%) relative to ST	0.25 (0.18-0.35)	<0.001	0.0016
DM Cocktail (Delta Tumor 20%) relative to ST	0.57 (0.43-0.76)	<0.001	3.2e-7
DM Cocktail (Delta Tumor 40 %) relative to ST		n.s. (0.166)	
DM Cocktail (Delta Tumor 10%) relative to DM Cocktail (Delta Tumor 5%)		n.s. (0.996)	
DM Cocktail (Delta Tumor 20%) relative to DM Cocktail (Delta Tumor 10%)	11.68 (5.61-24.32)	<0.001	7e-9
DM Cocktail (Delta Tumor 40%) relative to DM Cocktail (Delta Tumor 20%)	6.73 (3.74-12.11)	<0.001	4.3e-5
DM Ping-Pong Alternate Every Cycle (Delta Tumor 5%) relative to ST	0.31 (0.22-0.44)	<0.001	0.5
DM Ping-Pong Alternate Every Cycle (Delta Tumor 10%) relative to ST	0.26 (0.18-0.38)	<0.001	0.66
DM Ping-Pong Alternate Every Cycle (Delta Tumor 20%) relative to ST	0.50 (0.37-0.68)	<0.001	0.053
DM Ping-Pong Alternate Every Cycle (Delta Tumor 40 %) relative to ST	0.67 (0.50-0.90)	0.00795	4.2e-7

DM Ping-Pong Alternate Every Cycle (Delta Tumor 10%) relative to DM Ping-Pong Alternate Every Cycle (Delta Tumor 5%)		n.s. (0.397)	
DM Ping-Pong Alternate Every Cycle (Delta Tumor 20%) relative to DM Ping-Pong Alternate Every Cycle (Delta Tumor 10%)	1.97 (1.22-3.18)	0.00571	0.097
DM Ping-Pong Alternate Every Cycle (Delta Tumor 40%) relative to DM Ping-Pong Alternate Every Cycle (Delta Tumor 20%)		n.s. (0.0517)	
DM Ping-Pong on Progression (Delta Tumor 5%) relative to ST	0.09 (0.05-0.16)	<0.001	0.0018
DM Ping-Pong on Progression (Delta Tumor 10%) relative to ST	0.13 (0.08-0.22)	<0.001	0.045
DM Ping-Pong on Progression (Delta Tumor 20%) relative to ST	0.40 (0.29-0.55)	<0.001	0.82
DM Ping-Pong on Progression (Delta Tumor 40 %) relative to ST		n.s. (0.545)	
DM Ping-Pong on Progression (Delta Tumor 10%) relative to DM Ping-Pong on Progression (Delta Tumor 5%)		n.s. (0.356)	
DM Ping-Pong on Progression (Delta Tumor 20%) relative to DM Ping-Pong on Progression (Delta Tumor 10%)	3.20 (1.80-5.68)	<0.001	0.018
DM Ping-Pong on Progression (Delta Tumor 40%) relative to DM Ping-Pong on Progression (Delta Tumor 20%)	2.71 (1.71-4.28)	<0.001	0.022
FD Dose-Skipping/Drug Holiday (Delta Tumor 5%) relative to ST	1.50 (1.12-2.01)	0.00631	4.2e-8
FD Dose-Skipping/Drug Holiday (Delta Tumor 10%) relative to ST	1.65 (1.23-2.21)	<0.001	1.2e-7

FD Dose-Skipping/Drug Holiday (Delta Tumor 20%) relative to ST	1.45 (1.11-1.98)	0.00832	0.00015
FD Dose-Skipping/Drug Holiday (Delta Tumor 40 %) relative to ST	1.59 (1.18-2.12)	0.00196	1.6e-6
FD Dose-Skipping/Drug Holiday (Delta Tumor 10%) relative to FD Dose-Skipping (AT-2) (Delta Tumor 5%)		n.s. (0.484)	
FD Dose-Skipping/Drug Holiday (Delta Tumor 20%) relative to FD Dose-Skipping (AT-2) (Delta Tumor 10%)		n.s. (0.254)	
FD Dose-Skipping/Drug Holiday (Delta Tumor 40%) relative to FD Dose-Skipping (AT-2) (Delta Tumor 20%)		n.s. (0.51)	
FD Intermittent (Stop At 50% of Start) relative to ST	1.67 (1.25-2.24)	<0.001	1.6e-10
FD Intermittent (Stop At 80% of Start) relative to ST	1.45 (1.08-1.94)	0.0127	1.6e-8
FD Intermittent (Stop At 90% of Start) relative to ST		n.s. (0.154)	
FD Intermittent (Stop At 95% of Start) relative to ST		n.s. (0.0558)	
FD Intermittent (Stop At 80% of Start) relative to FD Intermittent (Stop At 50% of Start)		n.s. (0.0767)	
FD Intermittent (Stop At 90% of Start) relative to FD Intermittent (Stop At 80% of Start)		n.s. (0.13)	
FD Intermittent (Stop At 95% of Start) relative to FD Intermittent (Stop At 90% of Start)		n.s. (0.432)	

Table S6. Hazard ratios along with the 95% confidence intervals (c.i.), *p*-values, and test for proportionality of hazards *p*-values for Delta Dose Parameter. Abbreviations: n.s. is not significant, ST is standard treatment.

Comparison	Hazard Ratio (95% c.i.)	*p* value	Test for proportionality of hazards *p* value
DM Cocktail (Delta Dose 25%) relative to ST		n.s. (0.355)	
DM Cocktail (Delta Dose 50%) relative to ST	0.34 (0.24-0.47)	<0.001	1.2e-5
DM Cocktail (Delta Dose 75%) relative to ST	0.40 (0.29-0.54)	<0.001	3.3e-9
DM Cocktail (Delta Dose 50%) relative to DM Cocktail (Delta Dose 25%)	0.09 (0.04-0.18)	<0.001	1.2e-6
DM Cocktail (Delta Dose 75%) relative to DM Cocktail (Delta Tumor 50%)	1.74 (1.13-2.67)	0.0112	0.68
DM Ping-Pong Alternate Every Cycle (Delta Dose 25%) relative to ST	0.37 (0.26-0.53)	<0.001	0.79
DM Ping-Pong Alternate Every Cycle (Delta Dose 50%) relative to ST	0.46 (0.33-0.64)	<0.001	0.0018
DM Ping-Pong Alternate Every Cycle (Delta Dose 75%) relative to ST	0.39 (0.27-0.55)	<0.001	0.069
DM Ping-Pong Alternate Every Cycle (Delta Dose 50%) relative to DM Ping-Pong Alternate Every Cycle (Delta Dose 25%)		n.s. (0.306)	
DM Ping-Pong Alternate Every Cycle (Delta Dose 75%) relative to DM Ping-Pong Alternate Every Cycle (Delta Tumor 50%)		n.s. (0.451)	

DM Ping-Pong on Progression (Delta Dose 25%) relative to ST	0.13 (0.08-0.23)	<0.001	0.021
DM Ping-Pong on Progression (Delta Dose 50%) relative to ST	0.15 (0.09-0.25)	<0.001	0.00064
DM Ping-Pong on Progression (Delta Dose 75%) relative to ST	0.29 (0.20-0.44)	<0.001	0.085
DM Ping-Pong on Progression (Delta Dose 50%) relative to DM Ping-Pong on Progression (Delta Dose 25%)		n.s. (0.754)	
DM Ping-Pong on Progression (Delta Dose 75%) relative to DM Ping-Pong on Progression (Delta Tumor 50%)		n.s. (0.0795)	

Table S7. Hazard ratios along with the 95% confidence intervals (c.i.), *p*-values, and test for proportionality of hazards *p*-values for Treatment Vacation Parameter. Abbreviations: n.s. is not significant, ST is standard treatment.

Comparison	Hazard Ratio (95% c.i.)	*p* value	Test for proportionality of hazards *p* value
DM Cocktail (Treat Vacation at 10% of start) relative to ST	1.95 (1.45-2.61)	<0.001	0.00014
DM Cocktail (Treat Vacation at 50% of start) relative to ST	0.25 (0.18-0.35)	<0.001	0.0016
DM Cocktail (Treat Vacation at 80% of start) relative to ST	0.19 (0.13-0.26)	<0.001	2.1e-8
DM Cocktail (Treat Vacation at 50% of start) relative to DM Cocktail (Treat Vacation at 10% of start)	0.05 (0.02-0.11)	<0.001	0.019
DM Cocktail (Treat Vacation at 80% of start) relative to DM Cocktail (Treat Vacation at 50% of start)		n.s. (0.0966)	
DM Ping-Pong Alternate Every Cycle (Treat Vacation at 10% of start) relative to ST		n.s.(0.277)	
DM Ping-Pong Alternate Every Cycle (Treat Vacation at 50% of start) relative to ST	0.26 (0.18-0.38)	<0.001	0.66
DM Ping-Pong Alternate Every Cycle (Treat Vacation at 80% of start) relative to ST	0.19 (0.13-0.28)	<0.001	0.0031
DM Ping-Pong Alternate Every Cycle (Treat Vacation at 50% of start) relative to DM Ping-Pong Alternate Every Cycle (Treat Vacation at 10% of start)	0.25 (0.15-0.40)	<0.001	0.46
DM Ping-Pong Alternate Every Cycle (Treat Vacation at 80% of		n.s. (0.31)	

start) relative to DM Ping-Pong Alternate Every Cycle (Treat Vacation at 50% of start)			
DM Ping-Pong on Progression (Treat Vacation at 10% of start) relative to ST	1.38 (1.03-1.84)	0.0311	0.096
DM Ping-Pong on Progression (Treat Vacation at 50% of start) relative to ST	0.13 (0.08-0.22)	<0.001	0.045
DM Ping-Pong on Progression (Treat Vacation at 80% of start) relative to ST	0.16 (0.11-0.25)	<0.001	0.19
DM Ping-Pong on Progression (Treat Vacation at 50% of start) relative to DM Ping-Pong on Progression (Treat Vacation at 10% of start)	0.13 (0.07-0.24)	<0.001	0.73
DM Ping-Pong on Progression (Treat Vacation at 80% of start) relative to DM Ping-Pong on Progression (Treat Vacation at 50% of start)		n.s. (0.76)	

Table S8: Hazard ratios along with the 95% confidence intervals (c.i.), *p*-values, and test for proportionality of hazards *p*-values for various dose-modulation and fixed-dose adaptive therapy protocols relative to standard treatment (ST) under conditions when cells can undergo forward mutations only but no reverse mutations.

Protocol	Hazard Ratio (95% c.i.) relative to standard treatment	*p* value	Test for proportionality of hazards *p* value
DM Cocktail	0.33 (0.24-0.45)	<0.001	1.3e-7
DM Ping-Pong Alternate Every Cycle	0.46 (0.33-0.64)	<0.001	0.59
DM Ping-Pong on Progression	0.30 (0.20-0.44)	<0.001	0.38
FD Intermittent	1.79 (1.33-2.40)	<0.001	1.1e-12
FD Dose-Skipping/Drug Holiday	1.67 (1.24-2.24)	<0.001	9.1e-12

Table S9: Hazard ratios along with the 95% confidence intervals (c.i.), *p*-values, and test for proportionality of hazards *p*-values for various dose-modulation and fixed-dose adaptive therapy protocols relative to standard treatment (ST) under conditions when the doubling time of each cell type is increased by one order of magnitude relative to the default values.

Protocol	Hazard Ratio (95% c.i.) relative to standard treatment	*p* value	Test for proportionality of hazards *p* value
DM Cocktail	1.62 (1.21-2.17)	0.0011	0.016
DM Ping-Pong Alternate Every Cycle	0.88 (0.66-1.19)	n.s. (0.42)	0.33
DM Ping-Pong on Progression	1.00 (0.75-1.33)	n.s. (0.997)	0.018
FD Intermittent	1.00 (0.75-1.34)	n.s. (0.997)	1.7e-6
FD Dose-Skipping/Drug Holiday	0.31 (0.21-0.44)	<0.001	0.95
